# *Carinacuma umesi*, a new genus and species of bodotriid cumacean (Crustacea: Malacostraca: Peracarida) from shallow waters of the Maryland Coastal Bays, Mid-Atlantic region, USA

**DOI:** 10.7717/peerj.11740

**Published:** 2021-08-24

**Authors:** Andrés G. Morales-Núñez, Paulinus Chigbu

**Affiliations:** Department of Natural Sciences, University of Maryland Eastern Shore, Princess Anne, MD, United States of America

**Keywords:** Cumacea, Bodotriidae, Vaunthompsoniinae, *Carinacuma* gen. nov., *Carinacuma umesi* sp. nov., North America, Maryland, Mid-Atlantic coast

## Abstract

*Carinacuma*, a new bodotriid cumacean genus, is diagnosed to receive *Spilocuma watlingi* Omholt & Heard as its type species and *C*. *umesi* sp. nov., described from shallow waters (0.8 to 2.8 m) on the Mid-Atlantic coast of North America. *Carinacuma* gen. nov. has its closest affinities to the North American genera *Spilocuma* and *Mancocuma*, but can be distinguished from them and the other genera within the subfamily Vaunthompsoniinae by a combination of characters, including the presence of a dorsal carina or keel on pereonite 3 of the female, morphology of the antenna, absence of pleopods in the male, and the setation and segmentation of the uropods. *Carinacuma umesi* sp. nov., can be differentiated from its northern Gulf of Mexico cognate, *C*. *watlingi* comb. nov. by several characters, including: (1) maxilliped 3 carpus of female with inner margin bearing four to five simple setae, (2) uropod peduncle of female with inner margin bearing one sub-distal micro-serrate seta with single sub-terminal medial setule, (3) male antennule peduncle articles 1–2 sub-equal length, and (4) male antennule accessory flagellum slightly longer than basal article of main flagellum. A key to the five known males lacking pleopods within Vaunthompsoniinae is provided.

## Introduction

Members of the order Cumacea are small peracarid crustaceans, usually 1–35 mm in length, which are inhabitants of brackish and marine waters throughout the world (Heard, Roccatagliata & Petrescu, 2007; Gerken, 2018). Nine cumaceans species have been reported from the Chesapeake Bay (Zimmer, 1943; Zimmer, 1980; Wass, 1972). There are no publications, however, on the taxonomy and systematics of cumaceans in the Maryland Coastal Bays (MCBs), though information on the diversity and densities of cumaceans in the bays is available in several State reports (i.e., Llansó, Scott & Kelley, 2002; Llansó, Scott & Kelley, 2003; Llansó, Scott & Kelley, 2004; Llansó, Scott & Kelley, 2005; Llansó, Scott & Kelley, 2006; Llansó & Dew, 2010; Llansó, 2015).

Morales-Núñez & Chigbu (2016) reported a new Maryland distribution record for a bodotriid cumacean tentatively identified as “*Spilocuma watlingi* Omholt & Heard, 1979”. Upon further examination, however, this species was found to represent an undescribed species closely related to *Spilocuma watlingi*, which is endemic to the northeast Gulf of Mexico (GoM). The close similarity between *S. watlingi* and the new species from Maryland, and their distinctive differences with type species of *Spilocuma*, *S. salomani* Watling , 1977 necessitate the establishment of a new bodotriid genus within the bodotriid subfamily Vaunthompsoniinae Sars, 1878–1879 *sensu* Haye (2007).

The descriptions of these new taxa, including information on the abundance, distribution, and habitat of a new East coast species are presented herein.

## Materials and Methods

### Study area

**Figure 1:**
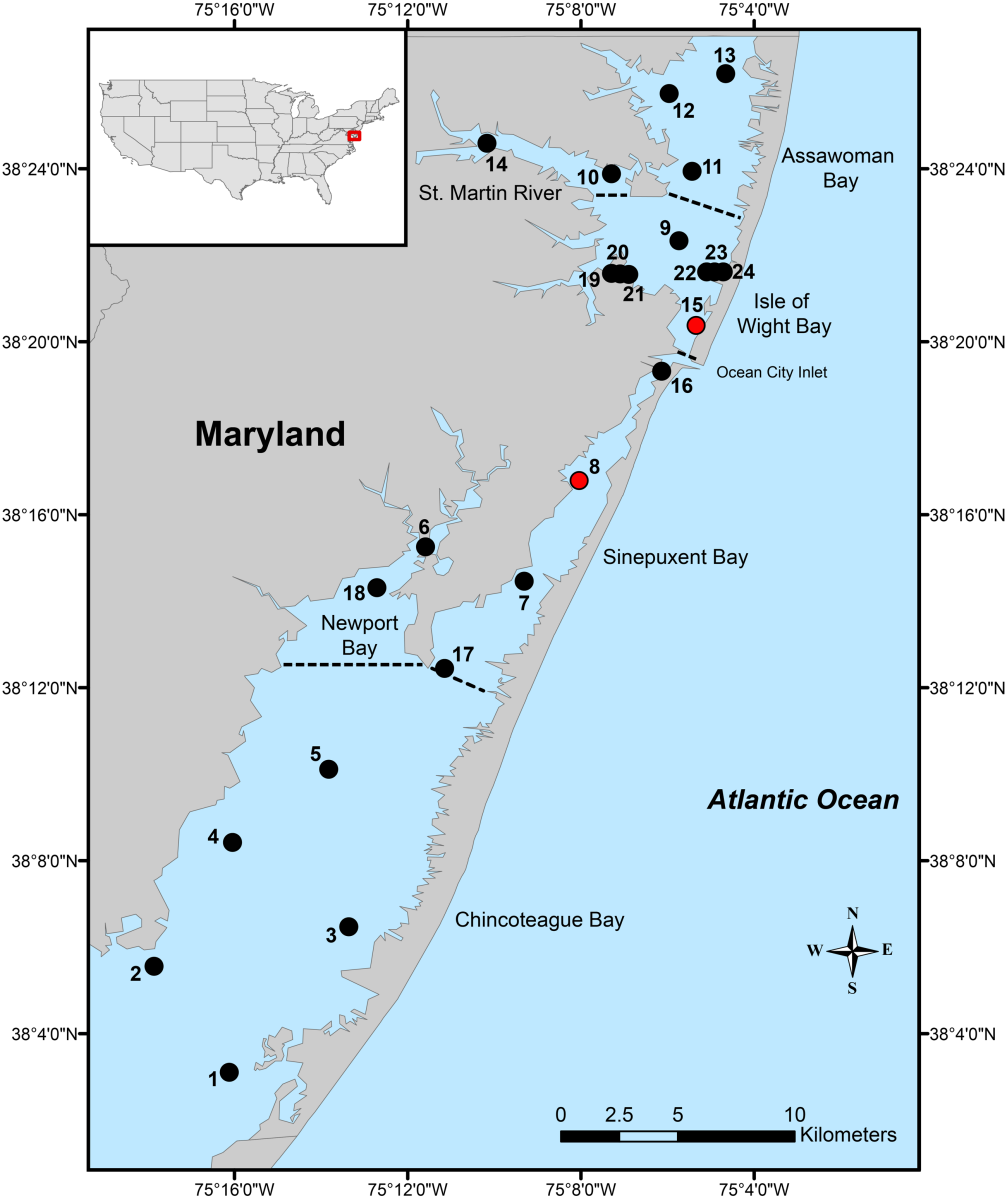
Local distribution. Map of Maryland Coastal Bays indicating the 24 stations sampled. Red circles indicate the only stations where *Carinacuma umesi* **gen. et sp. nov** was found; dotted lines separate bays/areas.

The Maryland Coastal Bays is a barrier-island system located on the eastern part of the Delmarva Peninsula in the eastern USA. The system consists of six principal lagoons/areas: Chincoteague Bay, Newport Bay, Sinepuxent Bay, Isle of Wight Bay, St. Martin River, and Assawoman Bay (Fig. 1).

### Sample collection and processing

Samples for this study were taken at 24 stations in the MCBs during May, June, August, and November from 2017 to 2019 (Fig. 1); although due to inclement weather conditions, samples were not collected from four stations in the Chincoteague Bay during May 2018. At each station, two sediment grab samples for biological analysis were taken using a 0.026 m^2^ stainless steel Van Veen grab (total area = 0. 052 m^2^). In the field, sediment samples for macroinvertebrates were passed through a 0.5 mm sieve screen (Eleftheriou & Mcintyre, 2005). After sieving, all the macroinvertebrates were fixed in 4% formalin with rose Bengal. In the laboratory, fixed macroinvertebrates were hand sorted, preserved in 70% ethanol, counted, and identified to the lowest possible taxonomic level. Additionally, one sample was collected at station-8 during a tryout of the new Van Veen benthic grab in August 2014; but sediment samples and water quality information were not collected.

One sediment sample was also collected for grain size and organic matter content analyses. Samples for grain size distribution and organic matter analyses were dried in a conventional oven at 105 °C and weighed until a constant weight was obtained. Sediment organic matter content was estimated by loss on ignition (LOI) method (Heiri, Lotter & Lemcke, 2001; Eleftheriou & Mcintyre, 2005). Grain size distribution was determined by sieving using the method described by Eleftheriou & Mcintyre (2005) and the United States Geological Survey (USGS) for Coarse Fraction Analysis (Gravel plus Sand). The particle size distribution and sorting of sediment were determined using the GRADISTAT v.9.1 software (Blott & Pye, 2001). Water quality data were collected *in situ* at each station using a YSI 6600 Multi-Parameter Water Quality Sonde and included water temperature, salinity, dissolved oxygen, and pH, which were all recorded at 0.3 m from the bottom.

**Figure 2:**
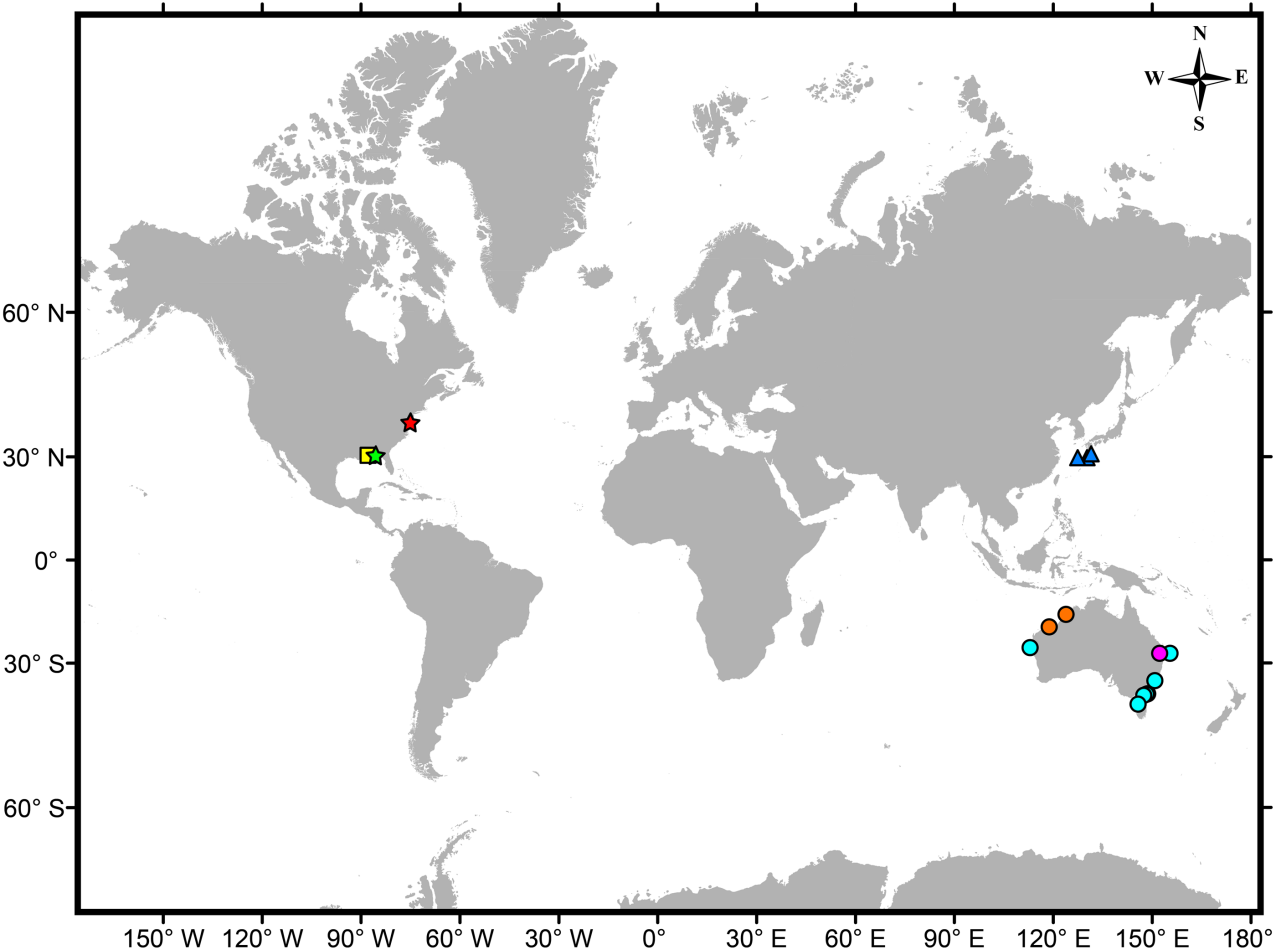
Map showing the worldwide distribution of known genera/species of males lacking pleopods within Vaunthompsoniinae. Circles –genus *Picrocuma*: *P*. *crudgingtoni* (magenta) (Watling & Gerken, 2020), *P*. *poecilotum* (cyan) (Hale, 1936; Tafe & Greenwood, 1996; GBIF Secretariat, 2020a; GBIF Secretariat, 2020b), *P*. *rectangularis* (orange) (Mühlenhardt-Siegel, 2003). Triangles –genus *Pseudopicrocuma*: *P*. *japonicum* (blue) (Akiyama, 2012). Star –genus *Carinacuma* **gen. nov.**: *C. umesi* **sp. nov.** (red) (this study), *C. watlingi* (light green) (Omholt & Heard, 1979; Modlin, 1992; GBIF Secretariat, 2020a; GBIF Secretariat, 2020b). Square –genus *Spilocuma*: *S*. *salomani* (yellow) (Watling, 1977; Modlin, 1992; GBIF Secretariat, 2020a; GBIF Secretariat, 2020b).

Specimens were dissected under an Olympus SXZ-16 stereomicroscope. Appendages were mounted on glass slides in glycerin and observed with an Olympus BX41 compound microscope, and drawings were made with a *camera lucida*. Drawings were re-drawn with a Wacom Cintiq Pro 13 - Creative Pen and touch display and Adobe Illustrator CC (2020). Figures were prepared with Adobe Illustrator CC (2020) and Photoshop CC (2020). Photographs were taken using an Olympus DP73 digital camera mounted on a stereomicroscope Olympus SXZ16 and all specimens were measured with CellSens dimensions 1.11 software (Olympus). Maps (Figs. 1, 2) were created using ArcGIS 10.4.2 software (University of Maryland Eastern Shore (UMES)).

The holotype and paratypes were measured and separated into three life-stage categories: (1) non-ovigerous females —without fully developed oöstegites, (2) ovigerous females —with fully developed oöstegites, and (3) male —smaller with strong antenna.

Type material has been deposited in the National Museum of Natural History, Smithsonian Institution, Washington, DC. All measurements were in millimeters. Total body length was measured from the frontal margin of the carapace to the posterior end of the pleon. Setal terminology follows that of Garm & Watling (2013).

### Nomenclatural acts

The electronic version of this article in Portable Document Format (PDF) will represent a published work according to the International Commission on Zoological Nomenclature (ICZN), and hence the new names contained in the electronic version are effectively published under that Code from the electronic edition alone. This published work and the nomenclatural acts it contains have been registered in ZooBank, the online registration system for the ICZN. The ZooBank LSIDs (Life Science Identifiers) can be resolved and the associated information viewed through any standard web browser by appending the LSID to the prefix http://zoobank.org/. The LSID for this publication is: [urn:lsid:zoobank.org:pub:3DF884AE-08A7-4CDD-B1D4-A7675ADC7F92]. The online version of this work is archived and available from the following digital repositories: PeerJ, PubMed Central and CLOCKSS.

## Results

### Systematics

**Table:** 

**Order Cumacea Krøyer, 1846**
**Family Bodotriidae T. Scott, 1901**
**Subfamily Vaunthompsoniinae Sars, 1878–1879**

**Type genus.***Vaunthompsonia* Bate, 1858

**Diagnosis.** After Haye (2007). Exopods occurring beyond first pair of pereopods. Females generally having three or more antennal articles. Uropod endopod generally bi-articulated.

**Genera.***Apocuma* Jones, 1973; *Bathycuma* Hansen, 1895; *Cumopsis* Sars, 1878–1879 (= *Heterocuma* Miers, 1879); *Gaussicuma* Zimmer, 1907; *Gephyrocuma* Hale, 1936; *Gigacuma* Kurian, 1951; *Glyphocuma* Hale, 1944; *Hypocuma* Jones, 1973; *Carinacuma* **gen. nov.**; *Leptocuma* Sars, 1873; *Mancocuma* Zimmer, 1943; *Paravaunthompsonia* Mühlenhardt-Siegel, 2008; *Picrocuma* Hale, 1936; *Pomacuma* Hale, 1944; *Pseudoleptocuma* Watling, 1977; *Pseudopicrocuma* Akiyama, 2012; *Pseudosympodomma* Kurian, 1954; *Scyllarocuma* Corbera, 2006; *Speleocuma* Corbera, 2002; *Spilocuma* Watling, 1977; *Sympodomma* Stebbing, 1912; *Vaunthompsonia*; *Zenocuma* Hale, 1944.

**Remarks.** Based on morphological phylogenetics analysis, Haye (2007), considered the subfamily Mancocumatinae Watling, 1977 as a junior synonym of the subfamily Vaunthompsoniinae. Further, Haye stated that the genus *Heterocuma* was synonymized with *Cumopsis* due to the strong molecular similarity (e.g., 79% bootstrap support) and lack of morphological differentiation (Haye, Watling & Kornfield, 2004; Haye, 2007). Notwithstanding, Luque & Gerken (2019) recently conducted a new morphological phylogenetic analysis of the family Bodotriidae in which they treated *Heterocuma* and *Cumopsis* as distinct genera.

### *Carinacuma* Morales-Núñez gen. nov.

*Spilocuma*.—Omholt & Heard, 1979 (in part).

**Type-species.***Carinacuma watlingi* (Omholt & Heard, 1979) **comb. nov.**

**Diagnosis.***Female*. *Carapace* lacking ridges or denticulations. Pereonite 3 with distinct dorsal keel or carina. Pereonite 5 with ventral keel or carina. *Antennule* peduncle having three articles; article 1 less than combined length of 2 and 3; peduncle articles 2 and 3 subequal; main flagellum subequal to peduncle article 3, main flagellum article 2 with two aesthetasc. *Antenna* with four articles. *Maxilliped 3* with basis having distal angle not produced. *Pereopod 1* without distal brush of setae on propodus and dactylus. Well-developed exopods present on pereopods 1–3; rudimentary on pereopod 4. *Uropod*: peduncle with inner margin bearing 1–6 simple or serrate setae with or without subterminal setule; endopod article 1 with inner margin having 4–7 bilaterally serrate seta with single sub-terminal medial setule, endopod article 2 with inner distal margin having one bilaterally serrate seta with single sub-terminal medial setule; exopod article 2 with inner distal margin bearing three micro-serrate setae with single sub-terminal medial setule.

*Male*. *Pereonite 3* lacking dorsal keel or carina. *Pereonite 5* with or without ventral keel or carina. *Antennule* with main flagellum tri-articulated. *Antenna* short not extending past carapace, modified for clasping female, peduncle stout with article-3 sparsely setose (lacking dense cluster of setae); flagella shorter than peduncle with modified “pad-like” setae on articles 10 and 11. *Pleopods* absent. *Uropod*: endopod having inner margin of article 1 bearing six bilaterally serrate seta with single sub-terminal medial setule; exopod article 2 having one micro-serrate seta with single sub-terminal setule medial seta on distal inner margin.

**Etymology.** From the Latin prefix (*Carina*) = keel, referring to the dorsal keel on the third pereonite of females + *Cuma* from which the ordinal name derives.

**Gender.** Feminine.

**Species.***Carinacuma umesi***sp. nov.**; *C. watlingi* (Omholt & Heard, 1979) **comb. nov.**;

**Distribution.** Known from shallow (0.5 to 3 m) coastal waters of the northwest Atlantic coastal region of North America (Maryland Coastal Bays, Mid-Atlantic region) and the northeast Gulf of Mexico (GoM), i.e., Alabama, Mississippi, and northwest Florida).

**Remarks.***Carinacuma***gen. nov**. appears to be most similar to the now monotypic genus *Spilocuma,* which is endemic to the northeast Gulf of Mexico, and to *Mancocuma* Zimmer, 1943*,* which is endemic to the Atlantic seaboard of North America. The female of *Carinacuma* is characterized by a dorsal keel or carina on pereonite 3, which immediately distinguishes it from these two genera, as well as, the other taxa within the subfamily Vaunthompsoniinae.

The male of *Carinacuma* also exhibits similarities to those of *Mancocuma* and *Spilocuma*. They all share specialized, short or relatively short prehensile “clasping” antennae, not extending posteriorly past the pereon, with stout peduncle (modified in varying degrees), and flagella curved ventromedially in a pre-copulatory, clasping orientation. The presence in the male of two pairs of reduced pleopods and a longer antenna flagellum with 20 or more articles in the less derived *Mancocuma* readily separates it from *Carinacuma* and *Spilocuma*.

The males of *Carinacuma* and *Spilocuma* are similar in having their antenna peduncles slightly longer than the flagella and lacking pleopods. *Carinacuma*, however, is distinguished from *Spilocuma* by having a: (1) uropod endopod article-1 with inner margin bearing six bilaterally serrate seta with single sub-terminal medial setule (14 in *Spilocuma*), (2) uropod endopod article-2 with inner margin having one bilaterally serrate seta with single sub-terminal medial setule (four in *Spilocuma*), and (3) uropod exopod with article 2 inner margin bearing one micro-serrate seta with single sub-terminal setule medial seta (two in *Spilocuma*).

As in the males of *Carinacuma* **gen. nov.** and *Spilocuma*, the genera *Picrocuma* from eastern Australia (Hale, 1936) and *Pseudopicrocuma* from southern Japan (Akiyama, 2012) lack pleopods. The number of pleopods on the other males within the subfamily Vaunthompsoniinae ranges from two to five pairs. There are two pairs on *Mancocuma* and *Speleocuma*; three pairs on *Pseudoleptocuma*; and five pairs on *Apocuma*, *Bathycuma*, *Cumopsis* (= *Heterocuma*), *Gaussicuma*, *Gephyrocuma*, *Gigacuma*, *Glyphocuma*, *Hypocuma*, *Leptocuma*, *Paravaunthompsonia*, *Pomacuma*, *Pseudoleptocuma*, *Pseudosympodomma*, *Sympodomma*, *Vaunthompsonia*. Males are unknown for the genera *Scyllarocuma* and *Zenocuma*.

At present the species representing *Carinacuma* and *Spilocuma* are known only from shallow-temperate and warm-temperate waters of the Atlantic and Gulf coasts of North America (Fig. 2). The other two genera are *Picrocuma* from western and eastern coasts of Australia (Fig. 2, Table 1) and *Pseudopicrocuma* from relatively deep-water (566–1679 m) in the northwestern Pacific Ocean (Fig. 2, Table 1). Based on other distinct morphological differences in their males, it is probable that the Vaunthompsoniinae genera from the Atlantic (*Carinacuma, Spilocuma*) and Pacific (*Picrocuma, Pseudopicrocuma*) are phylogenetically distant. In this case the mutual loss of pleopods would be due to homoplasy. These four genera and their species can be further distinguished using a dichotomous key presented herein.

**Table 1:** Recognized genera within Vaunthompsoniinae. Alphabetical listing of the 23 currently recognized genera within Vaunthompsoniinae, including information on distribution and depth range.

**Genus**	**Geographical area**	**Depth range (m)**
*Apocuma*	Atlantic Ocean New south Wales, Victoria, and Tasmania	587–2003 119–1500
*Bathycuma*	North Atlantic, Indian, and Pacific Oceans, Mediterranean Sea and off the coasts of South Africa	5000
*Cumopsis* (= *Heterocuma*)	East Atlantic and west Pacific Oceans and Mediterranean Sea	0–200
*Gaussicuma*	Southern Ocean Northwest Pacific Ocean	3400–4600 42–105
*Gephyrocuma*	Australian coast	0–75
*Gigacuma*	Indo-west Pacific	7–27
*Glyphocuma*	Southern Australian coast	0–100
*Hypocuma*	South Africa North Atlantic	400 1000–5000
*Carinacuma* **gen. nov.**	Northwest Atlantic Ocean (Gulf of Mexico and Mid-Atlantic region)	0.5–3
*Leptocuma*	Northeast Pacific Ocean Australian and South American	10 0–190
*Mancocuma*	Northwest Atlantic Ocean	0–18
*Paravaunthompsonia*	Red Sea	1446
*Picrocuma*	Southeast Indian Ocean and southwest Pacific Ocean (Australia)	1–4
*Pomacuma*	Australia and New Zealand	0–75
*Pseudoleptocuma*	Northwest Atlantic Ocean	15–24
*Pseudopicrocuma*	Northwest Pacific Ocean (Southern Japan)	566–1679
*Pseudosympodomma*	West Pacific Ocean (Tanzania) and Indo-west Pacific South Africa	0–4 85–370
*Scyllarocuma* ^a^	Southwest Pacific Ocean (New Caledonia, Australia)	650
*Speleocuma*	Northeast Atlantic Ocean (Tenerife, Canary Islands)	7 (cave complex)
*Spilocuma*	Northwest Atlantic Ocean Gulf of Mexico (Florida)	3
*Sympodomma*	West Indian Ocean, Indo-west and west Pacific, Australian coast, and South Pacific	115–1158
*Vaunthompsonia*	Mediterranean and red Seas, Indo-west and North Pacific, northwest tropical Atlantic (Caribbean Sea), Indian and Southern Oceans	0–280
*Zenocuma* ^a^	Southwest Pacific Ocean (Australia)	30–75

**Notes.**

a Indicates male unknown.

### *Carinacuma watlingi* (Omholt & Heard, 1979) comb. nov.

*Spilocuma watlingi*Omholt & Heard, 1979: 184–194, figs. 1–5.—Băcescu, 1988: 100.—Hopkins, Valentine & Lutz, 1989: 113, 115, 43e-f (key).—Rakocinski et al., 1991: 693.—Modlin, 1992: 83–90, fig 2., Table 1.— Rakocinski et al., 1996: 350 (Appendix 1).— Camp, 1998: 138 (List). —Heard, Roccatagliata & Petrescu, 2007: 35 (key), 105. —Heard & Roccatagliata, 2009: 1009 (check list).

**Type material.** United States National Museum. Not seen.

**Material examined.** Non-ovigerous ♂  (GCRL 05745), TBL 2.2 mm, station D4, Gulf of Mexico, Florida, USA, depth 1.5 m, sand, collector Sara E. LeCroy, 04 October 1989.—Adult ♂ (GCRL 05746), TBL 1.3 mm, station C6, (30° 18.37′N–87° 21.67′W), Gulf of Mexico, Florida, USA, depth 1.8 m, sand, collector Cathy Wooten, 04 December 1991.

**Diagnosis.** Modified from Omholt & Heard (1979). *Female*. *Antenna* second article without setae or spines. *Maxilliped 3* with carpus having 7–8 internal spines; propodus with four internal spines. *Uropod*: peduncle with inner margin bearing 3–6 sub-distal micro-serrate seta with single sub-terminal medial setule; endopod article 1 with inner margin having 4–6 bilaterally serrate seta with single sub-terminal medial setule.

*Male*. *Antennule* accessory flagellum shorter than basal article of main flagellum.

**Depth.** 1–1.8 m.

**Type locality:** Mobile Bay, southeast end of Dauphin Island, Alabama, 30°14′21″N, 88°04′42″W.

**Distribution.** Gulf of Mexico (Alabama, Mississippi, northwest Florida).

**Remarks.***Carinacuma watlingi***comb. nov.**, which is endemic to the northern GoM, is clearly congeneric and a cognate or sister species of the new species described herein from Maryland Coastal Bays.

### *Carinacuma umesi* Morales-Núñez sp. nov.

**Table:** 

(Figs. 3–13, 15K, 15M)

**Synonym:** *Spilocuma watlingi* Morales-Núñez & Chigbu, 2016

**Material examined.***Holotype*—Ovigerous ♂  (USNM 1658948), TBL 2.5 mm, station-15 (38°20.398′N–75°05.290′W), Isle of Wight Bay, Maryland, USA, depth 2.6 m, sand substrata, temperature 24.8 °C, salinity 32.8 PSU, DO 5.8 (mg/L), pH 7.8, collectors Andrés G. Morales-Núñez and Kayle Krieg, 10 August 2018.

*Paratypes*—Ovigerous female ♂  (dissected) (USNM 1658949), TBL 2.3 mm, station-15 (38°20.398′N–75°05.290′W), Isle of Wight Bay, Maryland, USA, depth 2.8 m, sand substrata, temperature 25.5 °C, salinity 30.9 PSU, DO 7.1 (mg/L), pH 7.6, collected by Andrés G. Morales-Núñez, 17 August 2017.—Ovigerous female ♂  (USNM 1658950), TBL 2.5 mm, station-8 (38°16.825′N–75°08.032′W), Sinepuxent Bay, Maryland, USA, depth 0.8 m, sand substrata, collectors Andrés G. Morales-Núñez and Alexis Jackson, 29 August 2014.—Non-ovigerous ♂  (USNM 1658951), TBL 2.3 mm, same collection data as holotype.—Adult ♂ (USNM 1658952), TBL 1.5 mm, same collection data as holotype.

**Diagnosis.***Female. Antenna* article 2 with sub- distal robust seta. *Maxilliped 3* with carpus having four simple setae on inner margin. *Uropod* peduncle with inner margin bearing one sub-distal micro-serrate seta with single sub-terminal medial setule. *Uropod* endopod article 1 inner margin with 6–7 bilaterally serrate seta with single sub-terminal medial setule.

*Male. Antennule* having accessory flagellum slightly longer than basal article of main flagellum.

**Etymology.** Named in honor of the University of Maryland Eastern Shore (UMES); the name is an acronym for UMES.

**Type locality.** Isle of Wight Bay, Station-15 (38°20.398′N–75°05.290′W), Maryland Coastal Bays, United States of America.

**Distribution.** Coastal shallow waters of Maryland Coastal Bays, Mid-Atlantic region –USA; Isle of Wight Bay, Station-15 (38°20.398′N–75°05.290′W) and Sinepuxent Bay Station-8 (38°16.825′N–75°08.032′W), at depths ranging from 0.8 to 2.8 m.

**Description.** Based on ovigerous female (USNM 1658949). Carrying nine eggs.

**Figure 3:**
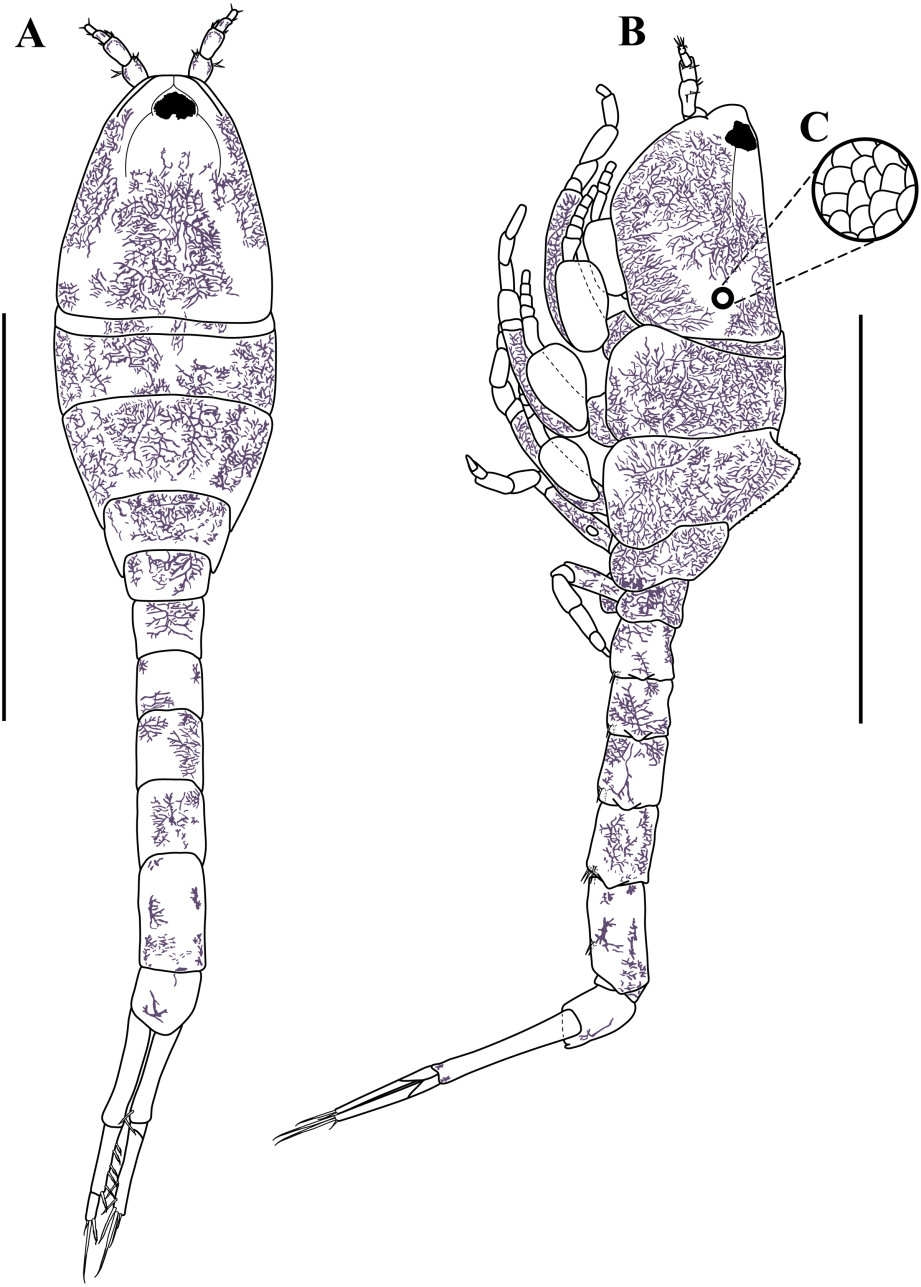
Habitus illustration (ovigerous female). *Carinacuma umesi***gen et sp. nov.**, Paratype ovigerous female: (A) dorsal view; (B) lateral view; (C) enlargement of the body scales. Scale bars = 1.0 mm for A–B.

*Body* (Figs. 3A–3C, 9A–9B). Length 2.3 mm, covered with purplish/brownish chromatophores dotted, integument covered by scales (Fig. 3C).

*Carapace* (Figs. 3A–3B, 9A–9B). About 25% of TBL, shorter than pereon, longer than wide; smooth, no ridges present; margins without any denticulation; laterally compressed anteriorly, not oviform; antennal notch shallow; pseudorostral lappets extend beyond frontal lobe and meet in mid-line.

*Pereon* (Figs. 3A–3B, 9A–9B). About 30% of TBL, shorter than pleon; all five segments visible in dorsal view (Fig. 3A); first pereonite visible only above lateral mid-line (Fig. 3B); second pereonite wide, and overriding pereonite 1 and carapace (Fig. 11B); third pereonite with distinct dorsal keel and overriding four pereonite (Fig. 3B); four pereonite overriding fifth pereonite; fifth pereonite with distinct ventral keel (Fig. 3B).

*Pleon* (Figs. 3A–3B, 8E, 9A–9B). About 45% of TBL, shorter than carapace and pereon together; pleonite 1 shortest; pleonite 5 about 1.9 times as long as wide, longest; pleonites 1–5 with four (two at each side) ventral setae; pleonite 6 asetose; pleonite 6 slightly longer than wide (Fig. 8E), shorter than uropod peduncle (Fig. 3A–3B, Fig. 8E), slightly extended past insertion of uropods (Fig. 8E). Pleopods absent.

**Figure 4:**
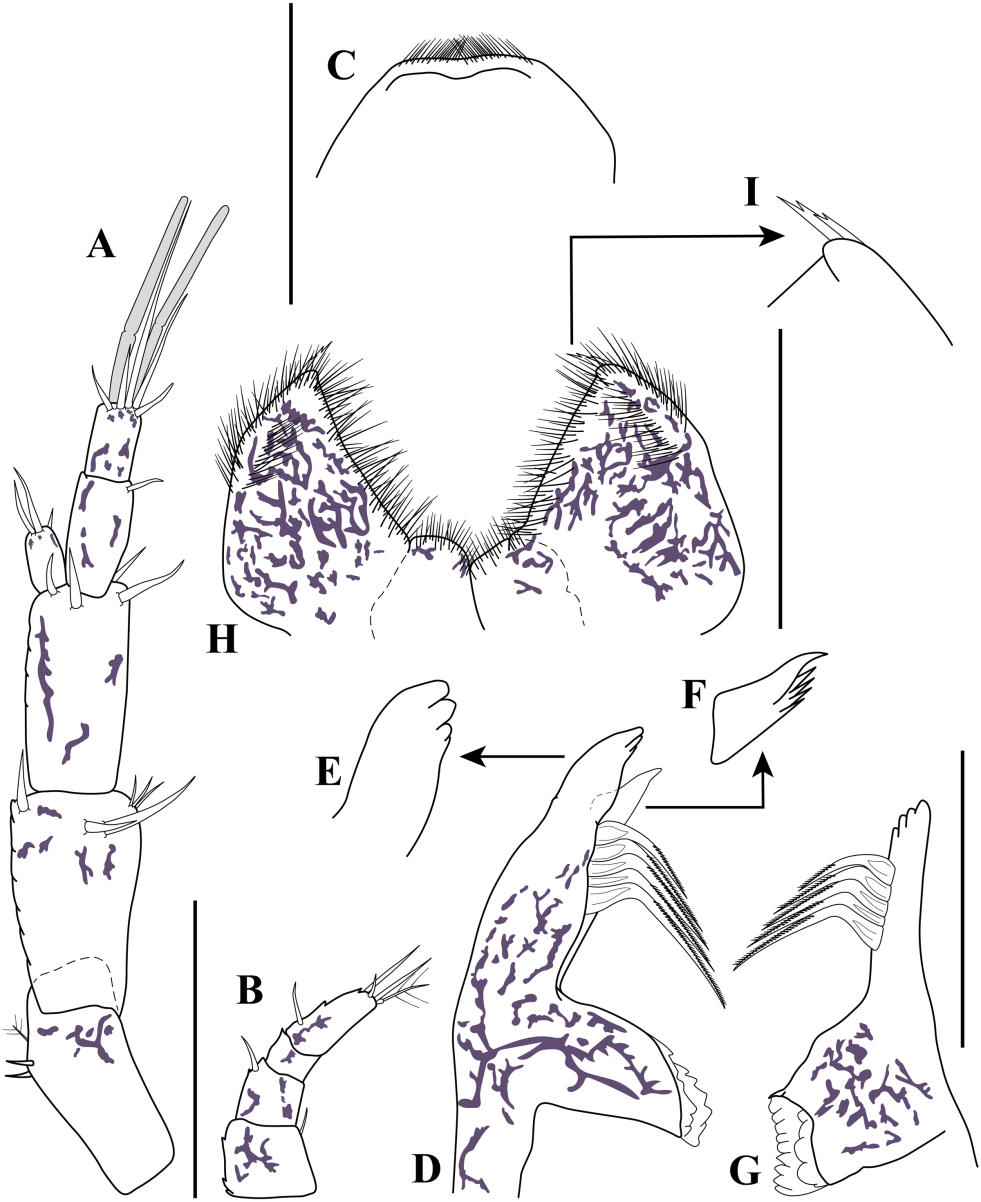
Antennule, antenna, and mouth parts illustrations. *Carinacuma umesi***gen. et sp. nov.**, Paratype ovigerous female: (A) antennule; (B) antenna; (C) labrum; (D) left mandible; (E) enlargement of left incisor; (F) enlargement of *lacinia mobilis*; (G) right mandible; (H) labium; (I) enlargement of labium tip. Scale bars = 0.1 mm for A–D, G, H.

**Figure 5:**
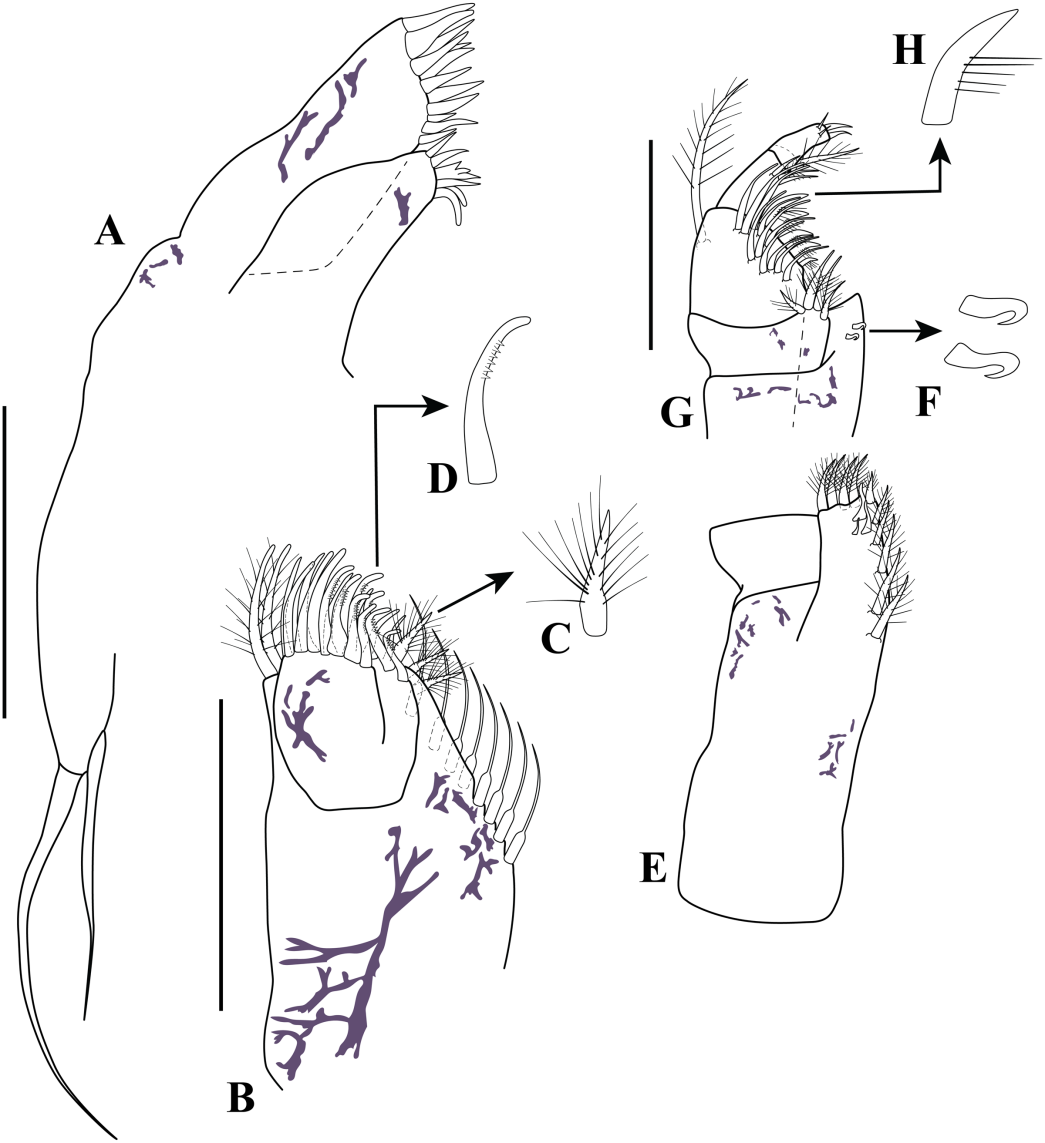
Mouth parts illustrations. *Carinacuma umesi***gen. et sp. nov.**, Paratype ovigerous female: (A) maxillule; (B) maxilla; (C) enlargement of pappose seta; (D) enlargement of microserrate seta; (E) maxilliped 1 (basis and endite); (F) enlargement of coupling hooks; (G) maxilliped 1 (ischium to dactylus); (H) enlargement of thick medially-setose seta. Scale bars = 0.1 mm for A–B, E, G.

*Antennule* (Fig. 4A). Peduncle with three articles; article 1 shortest with two sub-distal robust setae and one broom seta; article 2 twice as long as wide, distally with six simple setae (three small and three robust), article 3 sub-equal to article 2 length with four robust setae distally. *Main flagellum* bi-articulated; article 1 twice as long as wide with one distal simple seta, article 2 about 1.6 times as long as wide, distally with three robust setae, two simple setae of unequal lengths and two aesthetascs. *Accessory flagellum* uniarticulated; half-length basal article of main flagellum, distally with three robust setae of varying lengths.

**Figure 6:**
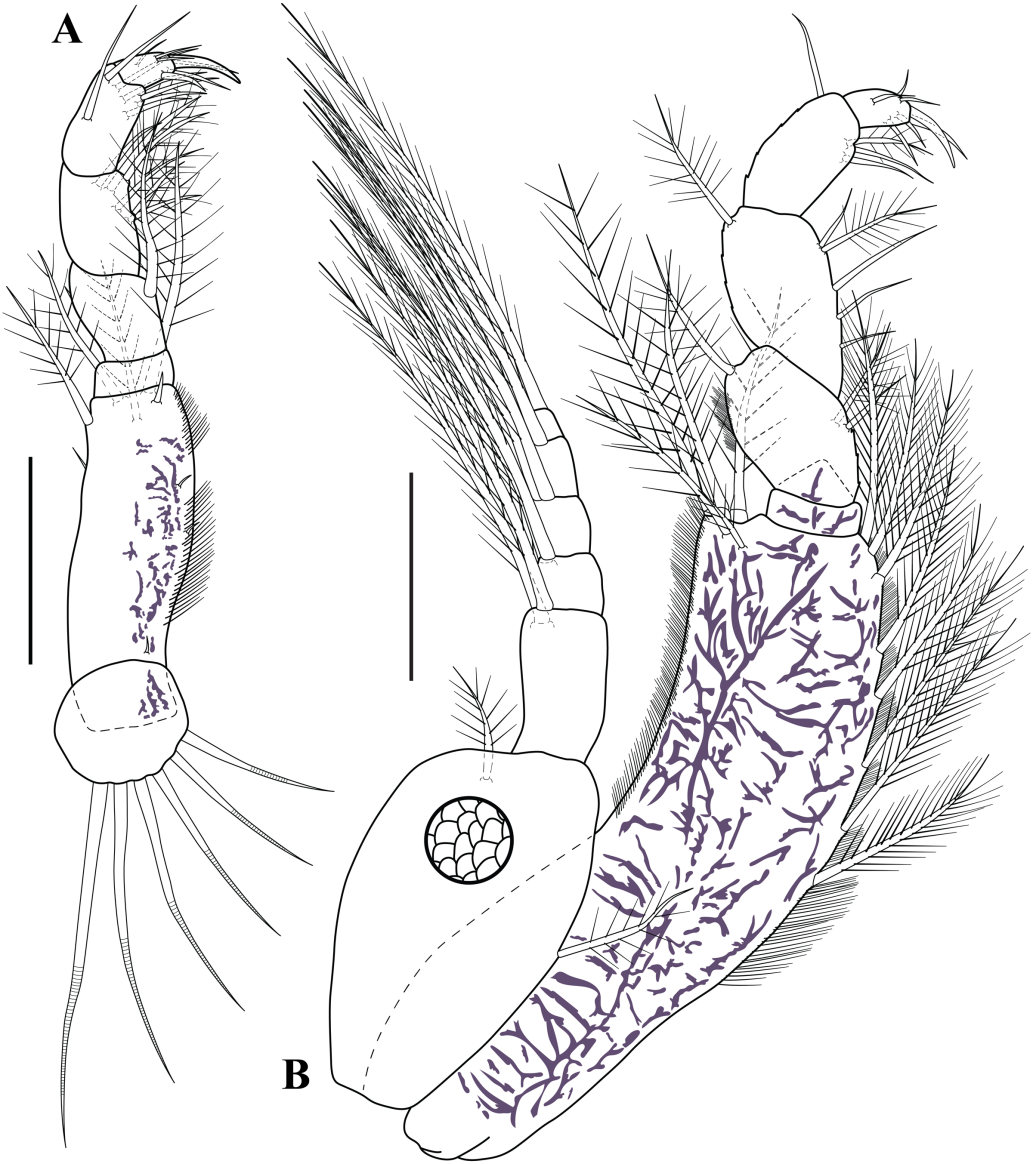
Maxillipeds illustrations. *Carinacuma umesi***gen. et sp. nov.**, Paratype ovigerous female: (A) maxilliped 2; (B) maxilliped 3. Scale bars = 0.1 mm.

**Figure 7:**
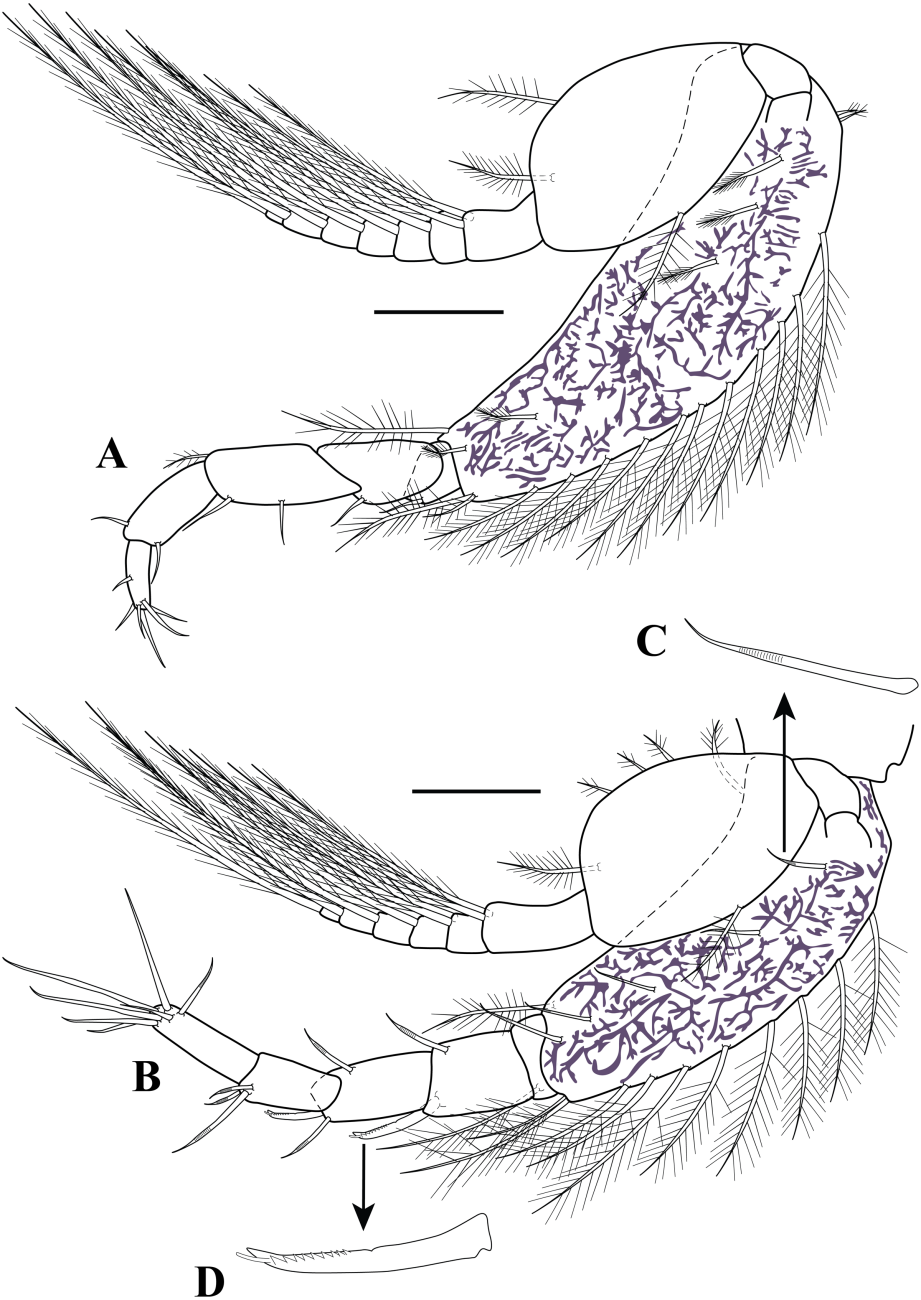
Pereopods and uropod. *Carinacuma umesi***gen. et sp. nov.**, Paratype ovigerous female: (A) pereopod 1; (B) pereopod 2; (C) enlargement of semi-annulate seta; (D) enlargement of strong serrated seta with single sub-terminal medial setule. Scale bars = 0.1 mm for A–B.

*Antenna* (Fig. 4B). Small, with four articles; article 1 wider than long with distal simple seta; article 2 sub-quadrate with sub-distal robust seta; article 3 wider than long, shortest, asetose; article 4 2.4 times longer than width, with sub-proximal robust seta, distally with one robust seta, two simple setae, and one broom seta.

*Labrum* (Fig. 4C). Distally setulate.

*Mandibles* (Figs. 4D–4G). Naviculoid form (not illustrated). Left and right mandible incisor with four denticles (Figs. 4D–4E, 4G); *lacinia mobilis* broad with five uneven teeth only present on left mandible (Fig. 4F); left and right setiferous lobe with five microserrate setae medially of different lengths (Figs. 4D, 4G). Molar process of left and right mandible similar, with grinding surface with well-developed micro-denticles (Figs. 4D, 4G).

**Figure 8:**
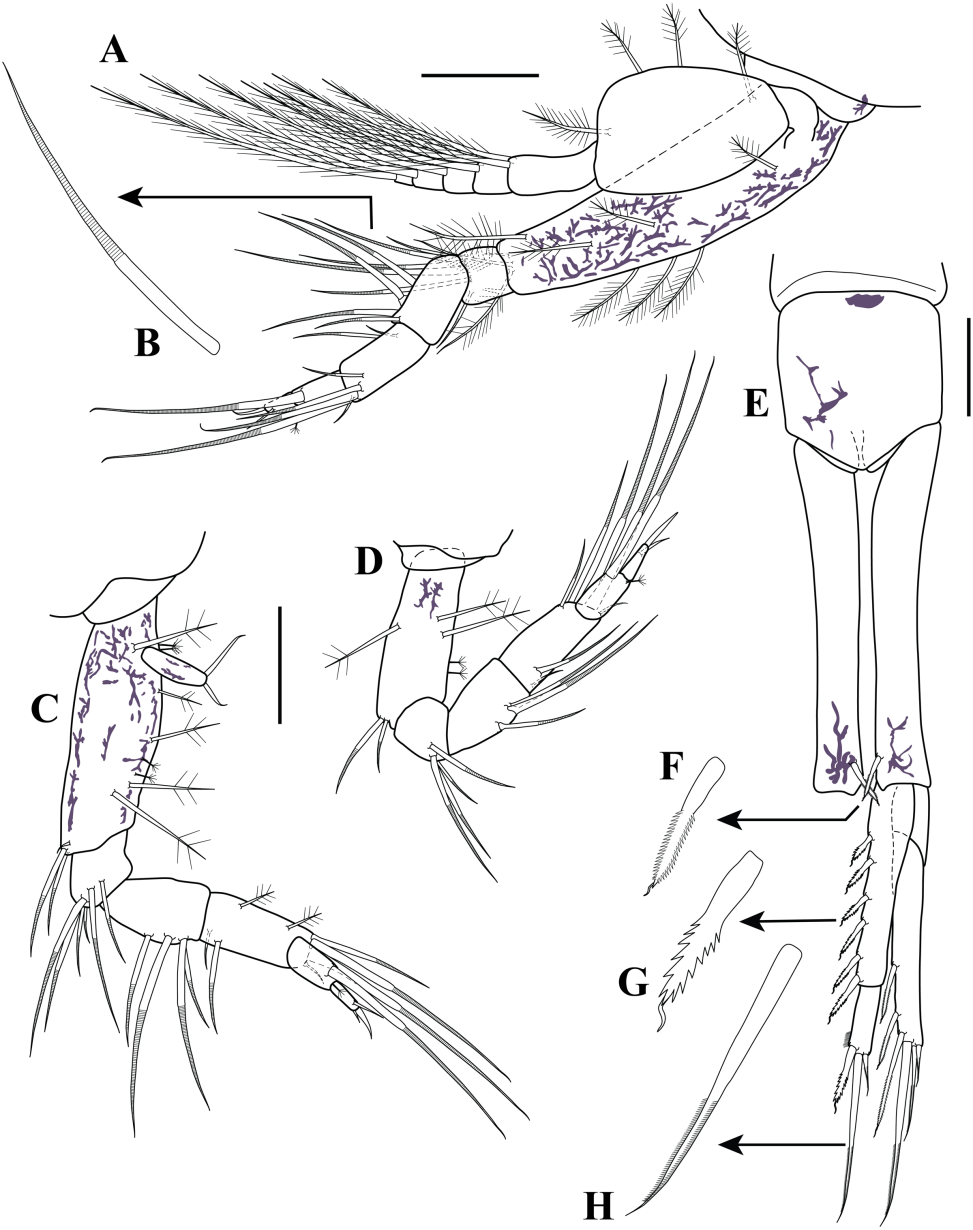
Pereopods and uropod. *Carinacuma umesi***gen. et sp. nov.**, Paratype ovigerous female: (A) pereopod 3; (B) enlargement of annulate seta; (C) pereopod 4; (D) pereopod 5; (E) Uropod; (F) micro-serrate seta with single sub-terminal setule medial seta; (G) serrate seta with single sub-terminal setule medial seta; (H) micro-serrate seta. Scale bars = 0.1 mm for A, C–E.

**Figure 9:**
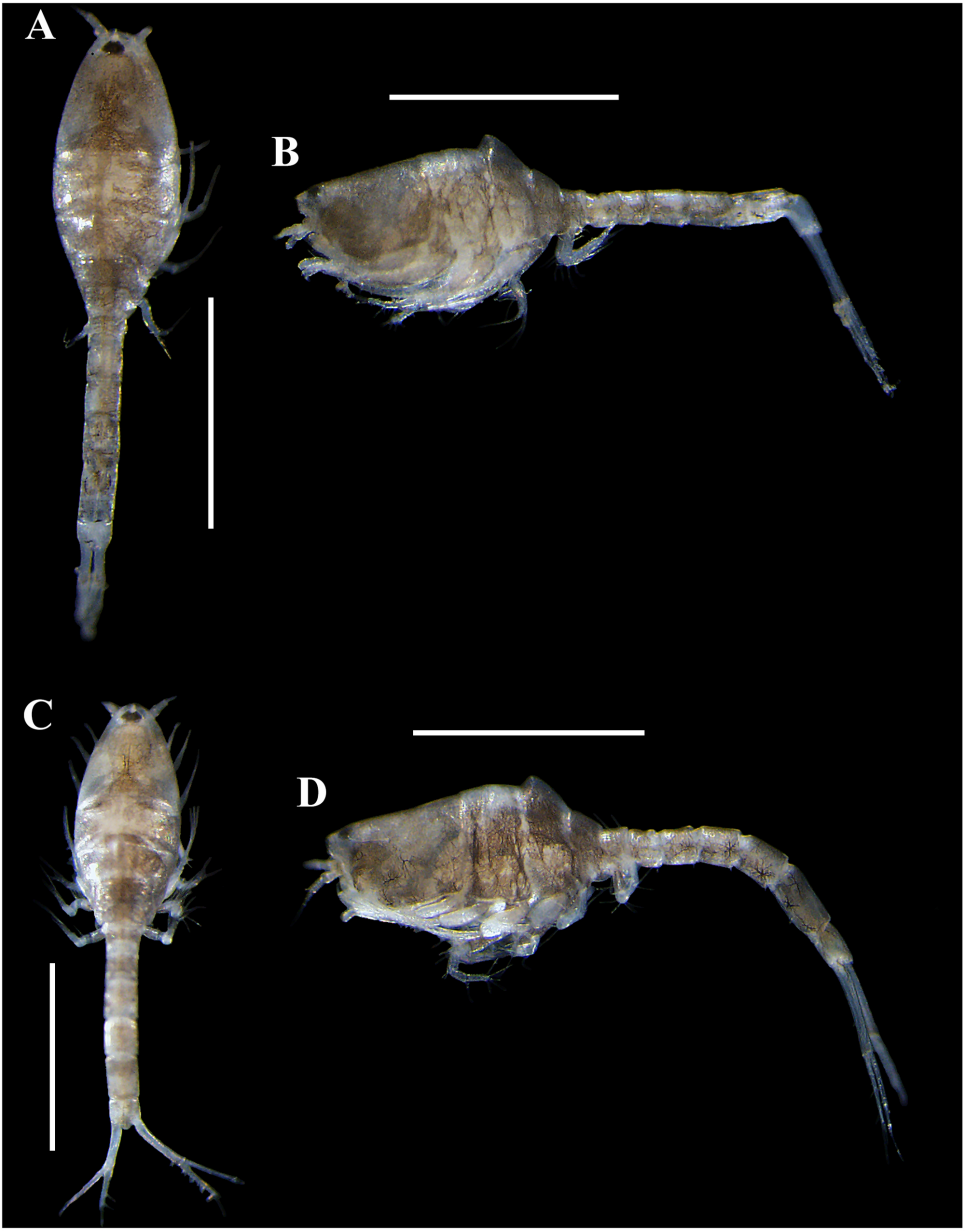
Pictures of habitus of *Carinacuma umesi* gen. et sp. nov. from Maryland Coastal Bays. Ovigerous female (dissected), paratype, TBL 2.3 mm: (A) dorsal view; (B) lateral view. Ovigerous venus , holotype, (USNM 1658948), TBL 2.5 mm: (C) dorsal view; (D) lateral view. Scale bars = 1.0 mm. Photos by A.G. Morales-Núñez.

*Labium* (Figs. 4H–4G). Inner margin setulose (Fig. 4H), mid-outer distal margin setulose (Fig. 4H), with two forked distal setae (Figs. 4H–4I).

*Maxillule* (Fig. 5A). With two endites; inner endite distal margin with five (two curved) simple setae and one trifurcate seta; outer endite distal margin with 12 simple setae; palp with two distal setae.

*Maxilla* (Figs. 5B–5D). With three endites; broad endite distal margin with five pappose (Fig. 5C) and five simple setae (Fig. 5B), and inner row of eight basally-swollen setae (Fig. 5B); inner narrow endite distal margin with three microserrate setae (Fig. 5D); distal narrow endite having distal margin with five microserrate and three simple setae, both narrow endites not extending past distal margin of broad endite (Fig. 5B).

**Figure 10:**
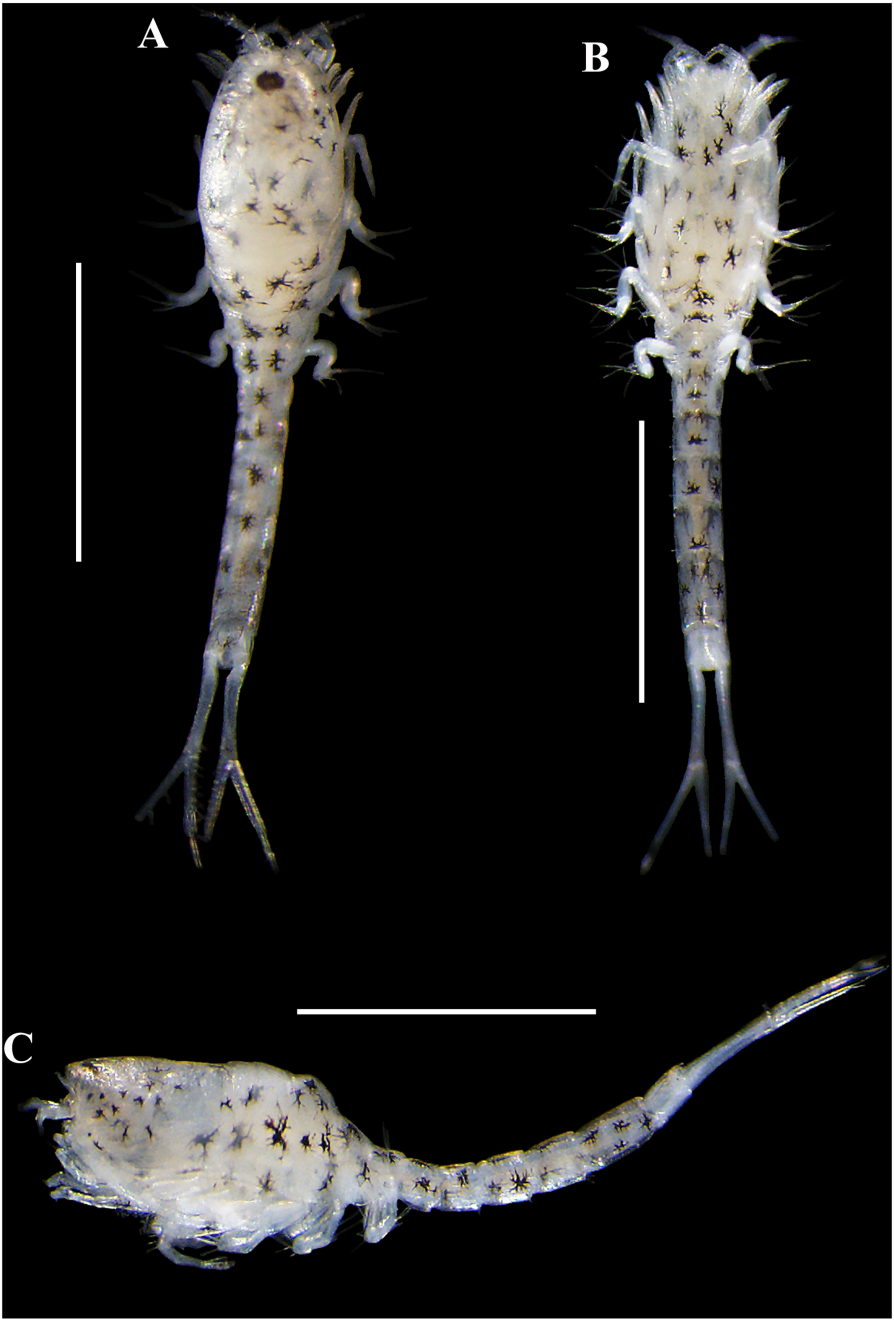
Pictures of habitus of *Carinacuma umesi* gen. et sp. nov. from Maryland Coastal Bays. Non-ovigerous female , paratype (USNM 1658951), TBL 2.3 mm: (A) dorsal view; (B) ventral view; (C) lateral view. Scale bars = 1.0 mm. Photos by A.G. Morales-Núñez.

**Figure 11:**
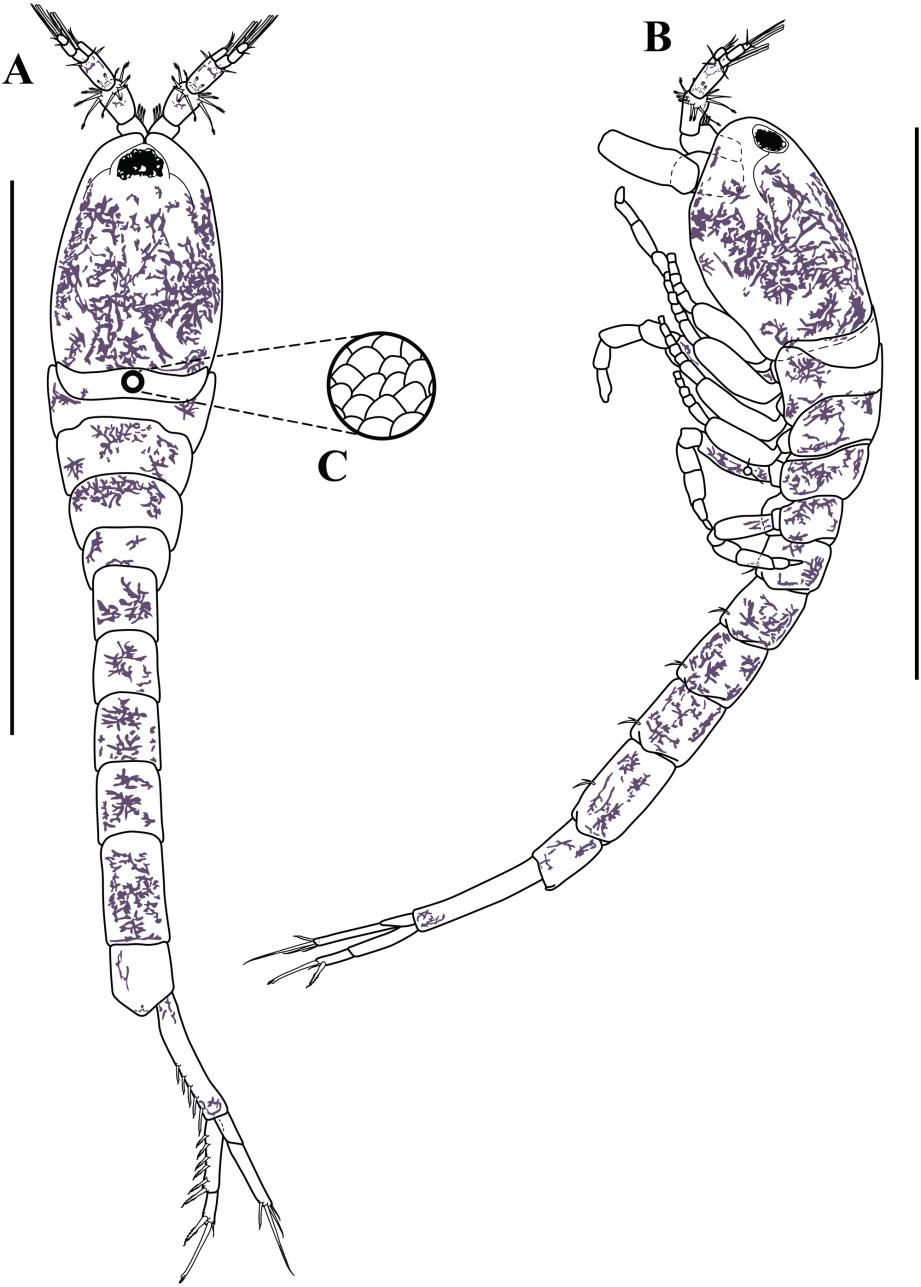
Habitus illustration (male). *Carinacuma umesi***gen et sp. nov.**, Paratype male: (A) dorsal view; (B) lateral view; (C) enlargement of the body scales. Scale bars = 1.0 mm for A–B.

*Maxilliped 1* (Fig. 5E–5H). Basis greater than combined lengths of remaining articles (Fig. 5E). Endite inner margin with row of five pappose setae (Fig. 5E), three subdistal simple setae (Fig. 5E), and two coupling hooks (Fig. 5F), distal margin with five pappose setae. Ischium absent. Merus wider than long, shortest, with four pappose setae distally. Carpus 2.4 times merus length, longest, inner margin with 11 simple setae and five thick medially-setose setae (Fig. 5H), sub-distal outer margin with plumose seta. Propodus 2.2 times as long as wide, sub-distal inner margin with four (two simple and two pappose) setae. Dactylus 2.8 times as long as wide, with four (two sub-distal and two distal) simple setae of unequal lengths.

*Maxilliped 2* (Fig. 6A). Coxa with six annulate setae. Basis 2.9 times as long as wide, shorter than combined lengths of other articles; inner margin finely setulose, with two small simple setae; sub-distal margin with one plumose seta, and distal margin with two plumose setae and simple seta; sub-distal outer margin with one small simple seta and one plumose seta. Ischium wider than long, shortest, asetose. Merus slightly shorter than carpus, with sub-distal inner plumose seta. Carpus 1.5 times as long as wide, inner margin with oblique row of four plumose setae and two (i.e., one mid and one sub-distal) plumose setae. Propodus 1.8 times as long as wide, twice dactylus length, inner margin with oblique row of four simple setae and two (one simple one plumose) sub-distal setae; mid-outer margin with one simple seta. Dactylus with inner margin bearing two sub-distal setae; distal margin with two (i.e., one simple and one strongly curved) setae; outer margin with simple setae mid-laterally and two simple setae sub-distolaterally.

*Maxilliped 3* (Fig. 6B). Basis 3.6 times as long as wide, greater than combined lengths of remaining articles, distal angle not produced; inner margin finely setulose, with eight plumose setae; outer margin finely setulose, with five plumose distally. Ischium wider than long, shorter than merus, asetose. Merus subequal to carpus; sub-distal inner margin with two plumose setae; mid-outer margin finely setulose with sub-distal plumose seta. Carpus shorter than propodus; inner margin with four simple setae of unequal lengths and one plumose seta; sub-distal outer margin with plumose seta. Propodus 1.6 times as long as wide; inner margin with three (one plumose and two simple) sub-distal setae; sub-distal outer margin with simple seta. Dactylus 1.6 times as long as wide; inner margin with two sub-distal setae; distal margin with two (i.e., one simple and one strongly curved) setae; outer margin with mid-laterally simple setae and two sub-distal laterally simple setae. Exopod 0.5 basis length; basal article unarmed; mid-inner margin with plumose seta; sub-distal margin with plumose seta; *flagellum* with five articles, each article bearing two plumose setae (not all illustrated).

*Pereopod 1* (Fig. 7A). Basis 3.0 times as long as wide, greater than combined lengths of remaining articles; inner margin with two sub-proximal broom setae and ∼13 plumose setae; mid-outer margin with five plumose setae; outer margin with plumose seta distally. Ischium shortest; inner margin with sub-distal simple seta. Merus 1.8 times as long as wide, shorter than carpus; inner margin with sub-distal simple seta. Carpus 2.2 times as long as wide, longer than propodus; inner margin with two (one mid and one sub-distal) simple setae; outer margin with one plumose seta distally. Propodus 2.1 times as long as wide; inner margin with sub-distal simple seta; outer margin with sub-distal simple seta. Dactylus 2.6 times as long as wide, shorter than propodus; distal margin with five simple setae; mid-outer margin with simple seta. Exopod 0.6 basis length; basal article unarmed; mid-inner margin with plumose seta; sub-distal margin with plumose seta; sub-distal outer margin with plumose seta; *flagellum* with seven articles, each article bearing two plumose setae (not all illustrated).

*Pereopod 2* (Figs. 7B–7D). Endopod shorter than first pereopod. Basis 2.7 times as long as wide, slightly shorter than combined lengths of remaining articles; inner margin with ten plumose setae; mid-outer margin with five semi-annulate setae (Fig. 7C); outer margin with two (one sub-proximal and one distal) plumose setae. Ischium wider than long, shorter than merus; inner margin with sub-distal simple seta. Merus 1.3 times as long as wide: inner margin with sub-distal strongly serrated seta with single sub-terminal medial setule (Fig. 7D); outer margin with sub-distal semi-annulate seta. Carpus 1.5 times as long as wide; inner margin with two (i.e., one semi-annulate and one sub-distal strongly serrated with single sub-terminal medial setule) setae; outer margin with sub-distal semi-annulate seta. Propodus twice as long as wide; outer margin with three sub-distal semi-annulate setae of different lengths. Dactylus 3.4 times as long as wide, longer than propodus; inner margin with three sub-distal semi-annulate setae of different lengths; distal margin with four simple setae of unequal lengths. Exopod 0.8 basis length; basal article unarmed; inner margin with mid-plumose seta; sub-distal margin with plumose seta; mid-outer margin with three plumose setae; *flagellum* with six articles, each article bearing two plumose setae (not all of them illustrated).

*Pereopod 3* (Figs. 8A–8B). Endopod shorter than first pereopod. Basis 3.4 times as long as wide, greater than combined lengths of remaining articles; inner margin with three plumose setae; mid-outer margin with three plumose setae; distal margin with three plumose setae; outer margin with sub-distal plumose setae. Ischium wider than long, shorter than merus; sub-distal margin with six annulate setae of varying lengths (Fig. 8B). Merus 1.7 times as long as wide; inner distal margin with four annulate setae. Carpus 2.2 times as long as wide; outer margin with sub-distal oblique row of five annulate setae of unequal lengths. Propodus 2.2 times as long as wide; sub-distal margin with one annulate seta; outer margin with distal broom seta. Dactylus twice as long as wide, shorter than propodus; sub-distal margin with simple seta; distal margin with simple seta. Exopod about 0.7 basis length; basal article unarmed; sub-distal margin with plumose seta; mid-outer margin with two plumose setae; *flagellum* with five articles, each article bearing two plumose setae (not all illustrated).

*Pereopod 4* (Fig. 8C). Shorter than first three pereopods. Basis 2.7 times as long as wide, shorter than combined lengths of remaining articles; inner distal margin with two annulate setae and one small simple seta; mid-outer margin with two (i.e., one sub-proximal and one sub-distal) plumose setae; outer margin with four broom setae and three plumose setae. Ischium wider than long, shorter than merus; sub-distal margin with six annulate setae of varying lengths. Merus 1.4 times as long as wide; inner distal margin with four annulate setae. Carpus 2.3 times as long as wide; inner margin with three (two sub-proximal and one distal) annulate setae of unequal lengths; outer margin with two (one sub-proximal and one sub-distal) plumose setae and sub-distal oblique row of five annulate setae of different lengths. Propodus 1.8 times as long as wide; distal margin with one broom seta; sub-distal outer margin with annulate seta. Dactylus 2.8 times as long as wide, shorter than propodus; distal margin with two (i.e., one small simple and one strongly curved) setae; sub-distal outer margin with simple seta. Rudimentary exopod, 3.5 times as long as wide; distal margin with two setae.

*Pereopod 5* (Fig. 8D). Basis 2.4 times as long as wide, shorter than combined lengths of remaining articles; inner margin with mid-plumose seta and two sub-distal annulate setae; mid-outer margin with two plumose setae; outer margin with two sub-distal broom seta. Ischium wider than long, shorter than merus; sub-distal margin with four annulate setae of varying lengths. Merus 1.6 times as long as wide; mid-inner margin with three annulate setae of unequal lengths. Carpus 1.9 times as long as wide; inner margin with three (two sub-proximal and one distal) annulate setae of unequal lengths; outer margin with sub-distal oblique row of four annulate setae of varying lengths. Propodus twice as long as wide; distal margin with one broom seta; sub-distal outer margin with annulate seta. Dactylus twice as long as wide, shorter than propodus; distal margin with one strongly curved seta; sub-distal outer margin with simple seta.

*Uropod* (Figs. 8E–8H). Peduncle 8.0 times as long as wide, longer than pleonite 6, longer than rami (excluding setae); inner margin with one sub-distal micro-serrate seta with single sub-terminal medial setule (Fig. 8F). Endopod biarticulated: article 1 7.0 times as long as wide, inner margin with six bilaterally serrate setae with single sub-terminal medial setule (Fig. 8G); article 2 3.1 times as long as wide; sub-distal inner margin finely setulose; distal margin with three setae (one bilaterally serrate seta with single sub-terminal medial setule and two micro-serrate setae (Fig. 8H)). Exopod bi-articulated: article 1 1.6 times as long as wide, asetose; article 2 8.0 times as long as wide, inner distal margin with three micro-serrate setae with single sub-terminal medial setule, distal margin with three micro-serrate setae of varying lengths.

**Non-ovigerous female (**without fully developed oostegites**).** Similar to ovigerous female except for having an underdeveloped dorsal keel on third pereonite.

*Body* (Figs. 10A–10B ). Length 2.3 mm.

*Carapace* (Figs. 10A–10B). About 25% of TBL.

*Pereon* (Figs. 10A–10B). About 30% of TBL. Third pereonite with an underdeveloped dorsal keel (Fig. 10C).

*Pleon* (Figs. 10A–10B). About 50% of TBL.

**Male.** Overall similar to adult females, except having: (1) smaller, more elongate, narrower body, (2) longer and robust antenna, (3) no dorsal keel, and (4) uropod peduncle with several setae. The only male obtained was grasping an ovigerous female when collected.

*Body* (Figs. 11A–11C, 13A–13B). Small, TBL 1.5 mm, covered with purplish/brownish chromatophores dotted, integument covered by scales (Fig. 11C).

*Carapace* (Figs. 11A–11B, 13A–13B). About 30% of TBL, longer than pereon, longer than wide; smooth, no ridges present; margins without any denticulation, laterally compressed anteriorly, not oviform.

*Pereon* (Figs. 11A–11B, 13A–13B). About 20% of TBL, shorter than pleon; all five segments visible in dorsal view (Fig. 11A); first pereonite visible only above lateral mid-line (Fig. 11B); second pereonite wide, and overriding pereonite 1 and carapace (Fig. 11B); third pereonite without dorsal keel (Fig. 11B); fifth pereonite with distinct ventral keel or carina (Fig. 3B).

**Figure 12:**
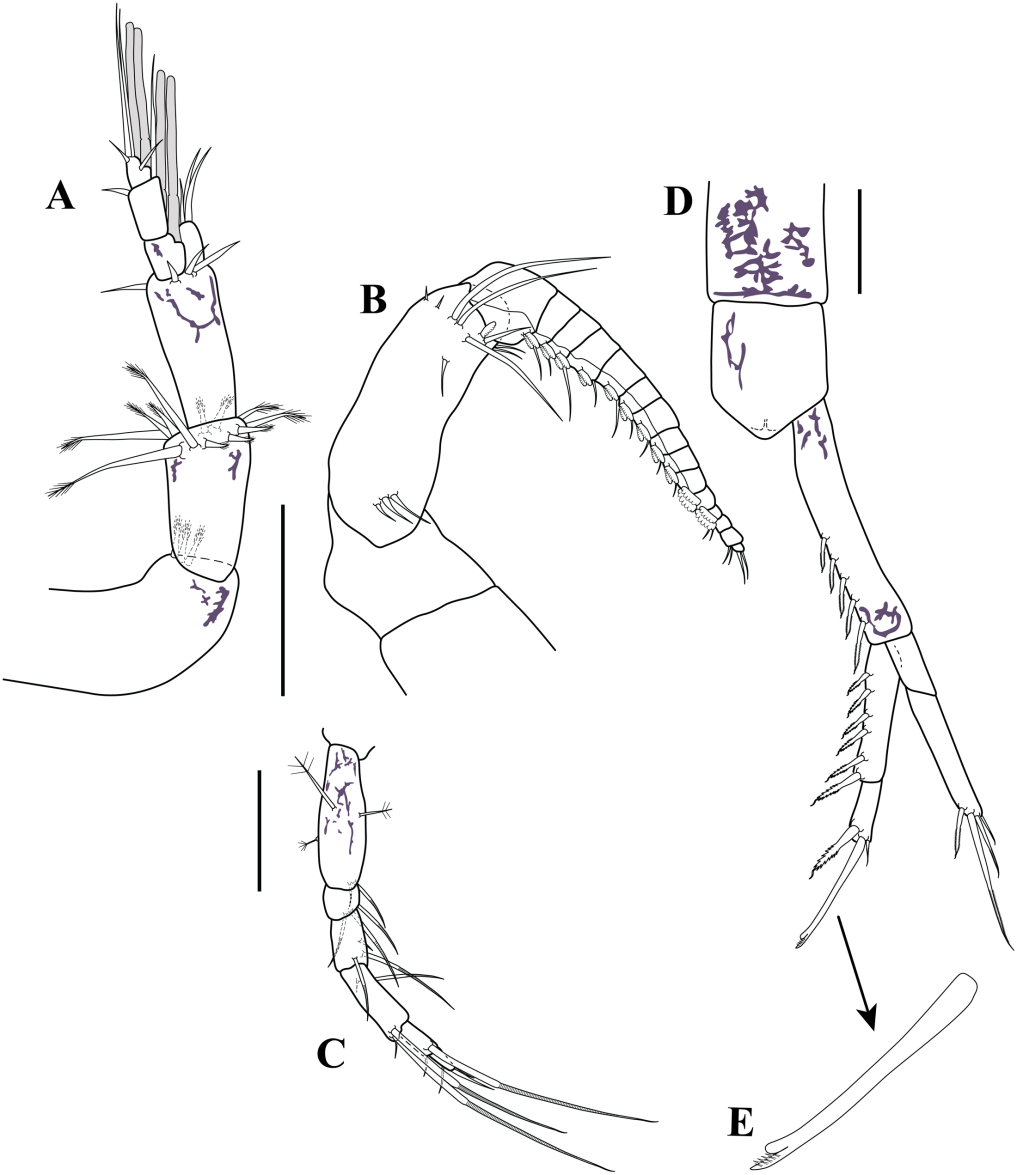
Male illustrations (parts). *Carinacuma umesi***gen. et sp. nov.**, Paratype male: (A) antennule; (B) antenna; (C) pereopod 5; (D) Uropod; (E) enlargement of bifid tip seta. Scale bars = 0.1 mm for A–D.

**Figure 13:**
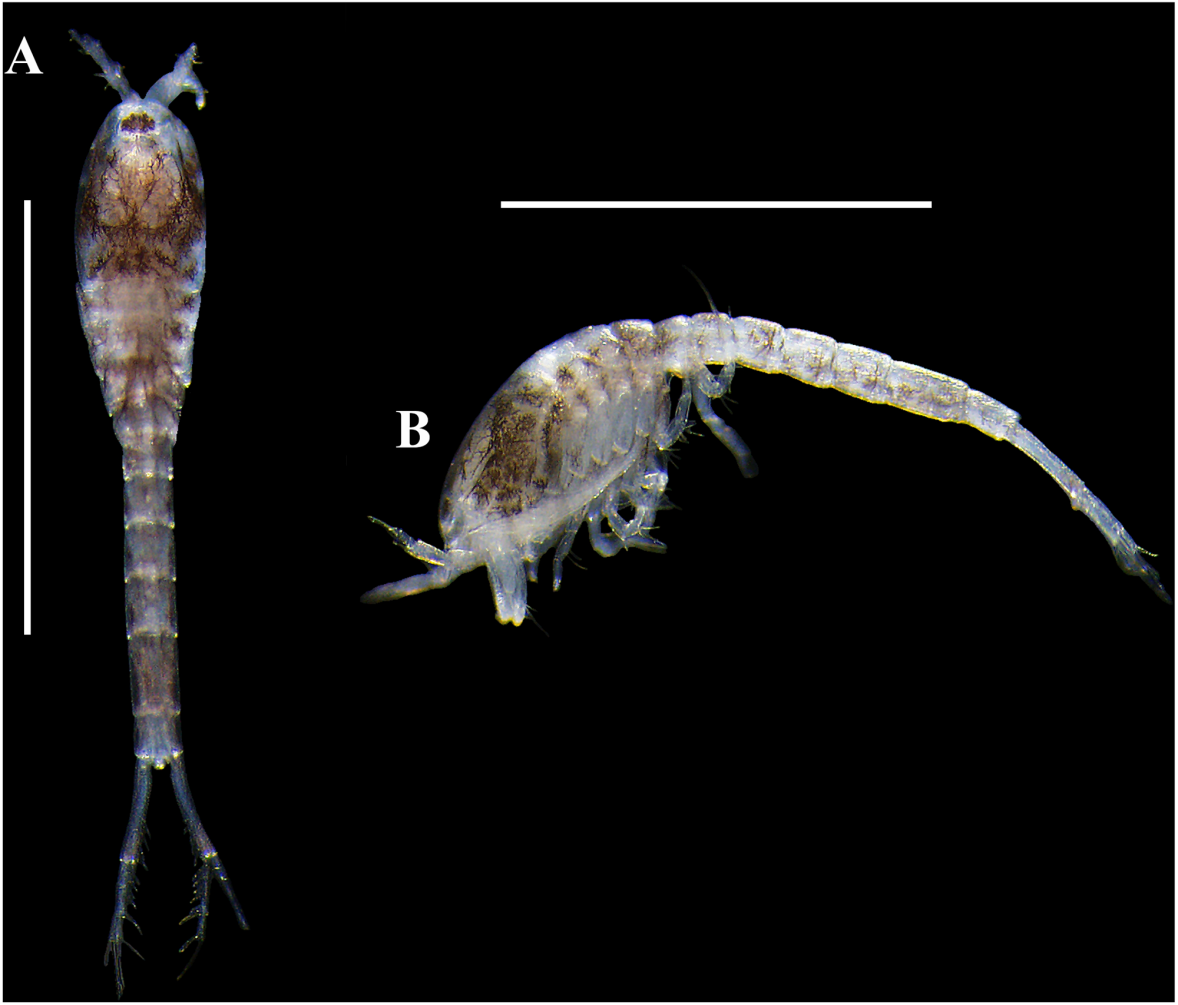
Pictures of habitus of *Carinacuma umesi* gen. et sp. nov. from Maryland Coastal Bays. Adult *male*, paratype, (USNM 1658952), TBL 1.5 mm: (A) dorsal view; B, lateral view. Scale bars = 1.0 mm. Photos by A.G. Morales-Núñez.

*Pleon* (Figs. 11A–11B, 12D, 13A–13B). About 50% of TBL, sub-equal to carapace and pereon together; pleonite 1 shortest; pleonite 5 about 1.8 times as long as wide, longest; pleonites 1–5 with four (two on each side) ventral setae; pleonite 6 asetose, longer than wide (Figs. 11A, 12D), shorter than uropod peduncle (Figs. 11A–11B, 12D), slightly extended past the insertion of the uropods (Fig. 8E). Pleopods absent.

*Antennule* (Fig. 12A). Peduncle with three articles; article 1 longest with three robust setae; article 2 twice as long as wide, with a crown of 12 robust setae sub-distally, article 3 sub-equal to article 2 length with four robust setae of unequal lengths distally. *Main flagellum* tri-articulated; article 1 about 1.2 times as long as wide with two aesthetascs; article 2 longest, 1.8 times as long as wide, distally with one robust seta and one simple seta; article 3 shortest, with two (one sub-distal and one distal) small robust setae, with two simple setae of unequal lengths, and two aesthetascs. *Accessory flagellum* uni-articulated; slightly longer than basal article of main flagellum, distally with three robust setae of varying lengths.

*Antenna* (Fig. 12B). Robust, geniculate, clasping form; peduncle with three articles; article 1–2 wider than long, asetose; article 3 longest, 2.8 times as long as wide, longer than article 1–2 combined, with three setae sub-proximally, nine setae of varying lengths sub-distally, and one flattened granulated adhesive pad on distal margin. *Flagellum* with 15 articles; articles 1–11 each with two flattened granulated adhesive pads, being larger on the articles 10–11 and passing next article; article 12–15 lacking pads; articles 1 −11 with one seta distally; articles 12 and 14 with two setae distally; article 13 asetose; article 15 terminating in two setae.

*Pereopod 5* (Fig. 12C). Basis 3.1 times as long as wide, shorter than combined lengths of remaining articles; inner margin with mid-plumose seta and two sub-distal annulate setae; mid-outer margin with one plumose seta; outer margin with one broom seta. Ischium wider than long, shorter than merus; sub-distal margin with three annulate setae of varying lengths. Merus 1.7 times as long as wide; mid-inner margin with three annulate setae of unequal lengths. Carpus 2.5 times as long as wide; sub-proximally inner margin with one annulate seta; sub-proximally mid-outer margin with one small simple seta; distal outer margin with one seta and two annulate setae of varying lengths. Propodus twice as long as wide; distal inner margin with one seta; sub-distal inner margin with annulate seta. Dactylus twice as long as wide, shorter than propodus; distal margin with one strongly curved seta; sub-distal outer margin with simple seta.

*Uropod* (Figs. 12D–12E). Peduncle 5.6 times as long as wide, longer than pleonite 6, longer than rami (excluding setae); inner margin with five sub-distal micro-serrate setae with single sub-terminal medial setule (Fig. 12D). Endopod bi-articulated: article 1 about 4.9 times as long as wide, inner margin with six bilaterally serrate setae with single sub-terminal medial setule (Fig. 12D); article 2 about three times as long as wide; distal margin with three (one bilaterally serrate seta with single sub-terminal medial setule, one long distal bifid tip (one tip rounded and one tip serrate, Fig. 12E), and one small micro-serrate) setae (Fig. 12D). Exopod bi-articulated: article 1 twice as long as wide, asetose; article 2 5.3 times as long as wide, inner distal margin with one micro-serrate seta with single sub-terminal medial setule, distal margin with three micro-serrate setae of varying lengths.

**Intraspecific variation.** Although only five specimens of *Carinacuma umesi* **sp. nov**. were available for the study, they exhibited some degree of variations among the individuals examined (Table 2) including: (1) the number of setae on the inner margin of the carpus of maxilliped 3 varied from four to five (holotype, USNM 1658948), (2) the number of setae on the sub-distal inner margin of the carpus and propodus varied from one to two (holotype, USNM 1658948), respectively, and (3) uropod peduncle with 6–7 (holotype, USNM 1658948) serrate setae with single sub-distal micro-serrate seta with single sub-terminal medial setule on inner margin.

**Size distribution.** Body sizes of *Carinacuma umesi* are presented in Table 2. Non-ovigerous female TBL 2.3 mm (Fig. 10). Ovigerous females ranged in size from 2.3 mm to 2.5 mm (*n* = 3) (Fig. 9). Male TBL 1.5 mm (Fig. 13).

**Coloration.** Habitus of non-ovigerous female  presented a whitish coloration with black chromatophores dotted (Fig. 10). Meanwhile, habitus of ovigerous females  and male presented a darker coloration with purplish/brownish chromatophores dotted (intensity can vary) (Figs. 9 and 13); color in male is more intense (Fig. 13). Following preservation in 70% ethanol for almost six years, the ovigerous female  collected in 2014 retained the coloration pattern, as did the other specimens. The exopods in all the specimens examined lack coloration.

**Figure 14:**
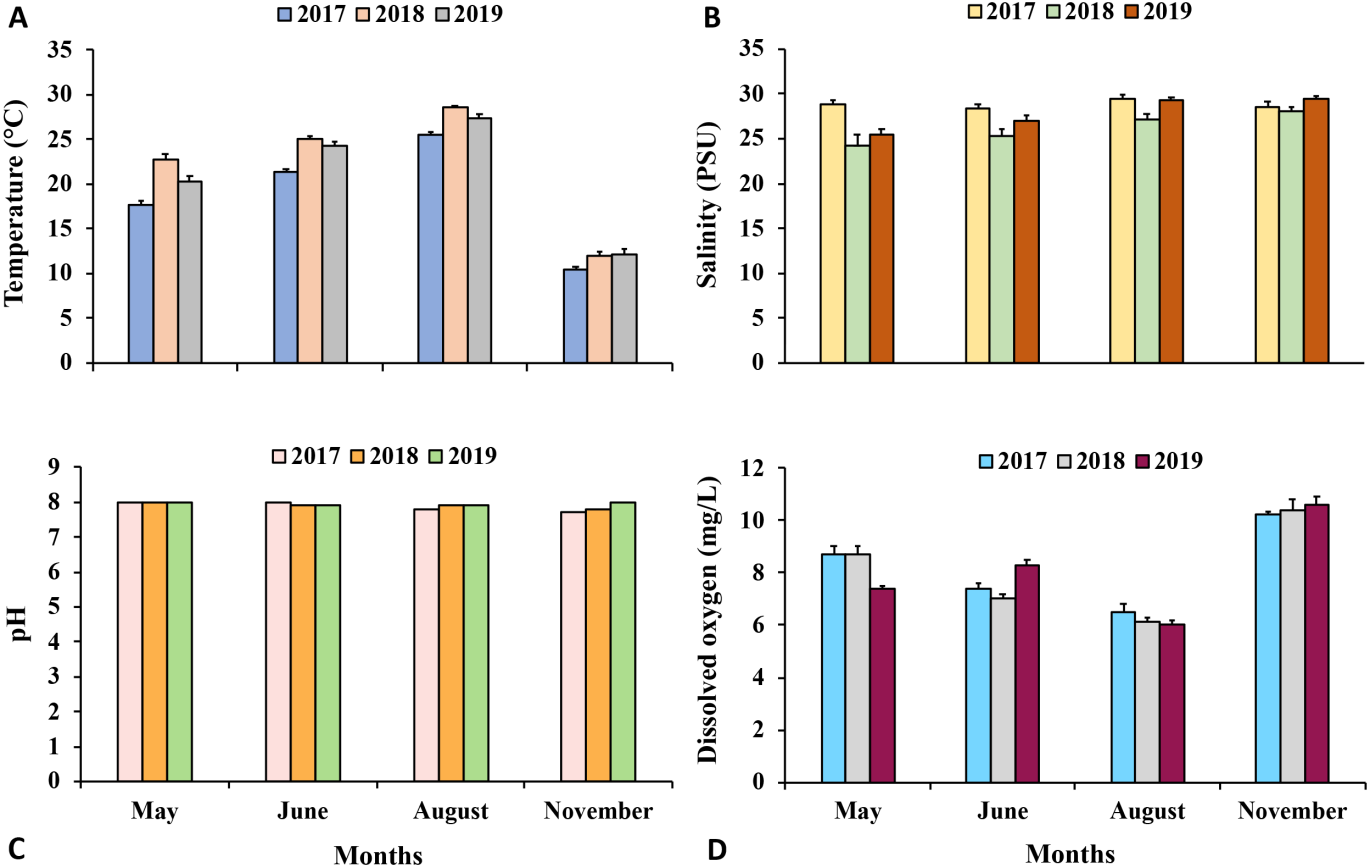
Environmental parameters. Mean values of environmental parameters measured at 24 stations per month in Maryland Coastal Bays during 2017–2019. Data are means ± SE. A, temperature; B, salinity; C, pH; D, DO.

**Ecological notes.** A total of five individuals of *Carinacuma umesi* were found in the MCBs. Specimens of *C*. *umesi* were only found at two of the 24 stations along the bays (Fig. 1) and were collected from sandy bottoms with, very well sorted, fine sand substrata having low organic content 0.4% ±0.1. Water temperature varied from 24.8–25.5 °C, salinity ranged from 30.9–32.8 PSU, pH varied from 7.6–7.8, and DO ranged from 5.8–7.1 (mg/L). The physicochemical parameters of the surrounding waters where *C. umesi* was collected are presented in Fig. 14.

**Figure 15:**
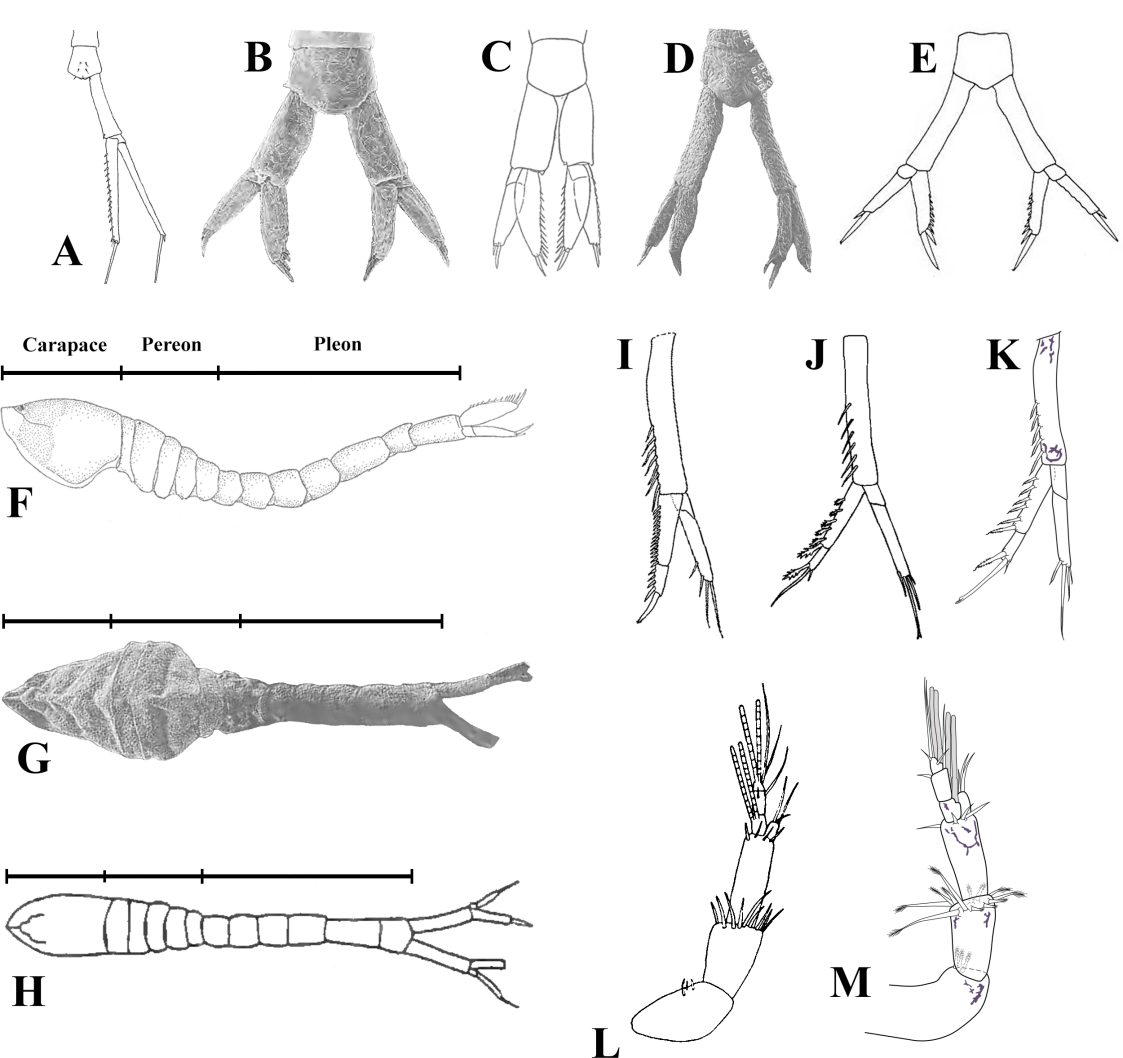
Illustrated key. Uropods: (A) *Pseudopicrocuma japonicum*; (B) *Picrocuma crudgingtoni*; (C) *P*. *poecilotum*; (D) *P*.* poecilotum*; (E) *P*.*rectangularis*; (I) *Spilocuma salomani*; (J) *Carinacuma watlingi* **comb. nov.** K, *C. umesi* **sp. nov.** Male habitus: (F) *Picrocuma poecilotum*, lateral view; (G) *P*. *poecilotum*, dorsal view; (H) *P*. *rectangularis*, dorsal view. Antennules: (L) *Carinacuma watlingi* **comb. nov.**; (M) *Carinacuma umesi* **sp. nov.** Figures modified from: A Akiyama (2012); B, D, G, Tafe & Greenwood (1996); C, F, Hale (1945); E, H, Mühlenhardt-Siegel (2003); I, Watling (1977); J, L, Omholt & Heard (1979); K, M, Morales-Núñez and Chigbu (this study).

Others peracarids co-occurring with the new species included: tanaidaceans (*Tanaissus* sp. Norman & Scott, 1906); amphipods (*Acanthohaustorius intermedius* Bousfield, 1965; *Bathyporeia parkeri* Bousfield, 1973; *Parahaustorius longimerus* Bousfield, 1965; *P*. *wigleyi* Bousfield, 1965); and cumaceans (*Mancocuma* cf. *altera* Zimmer, 1943 and *Oxyurostylis smithi* Calman, 1912).

**Table 2:** Morphological characters within *Carinacuma umesi*. Total body length (TBL) and comparison of some morphological features of sexual stages of *Carinacuma umesi* **sp. nov.** population.

**Stages**	**TBL (mm)**	**Carapace length (mm)**	**Pereon length (mm)**	**Pleon** length (mm)	**No. of micro-serrate seta with sub-terminal medial setule on inner margin of the uropodal peduncle**	**No. of serrate setae with single sub-terminal medial setule on inner margin of the uropodal endopod article 1**
**Non-ovigerous female (with oöstegites)**						
1 (Paratype, USNM: 1658951)	2.3	0.56	0.61	1.11	1	6
**Ovigerous females**						
1 (Holotype, USNM: 1658948)	2.5	0.57	0.74	1.16	1	7
2 (Paratype, dissected, USNM: 1658949)	2.3	0.59	0.69	1.06	1	6
3 (Paratype, USNM: 1658950)	2.5	0.55	0.78	1.17	1	6
**Male**						
1 (Paratype, USNM: 1658952)	1.5	0.41	0.33	0.74	5	6

**Remarks.** The female of *Carinacuma umesi* **sp. nov.** specifically differs from that of its GoM congener *C. watlingi* by having: (1) maxilliped 3 carpus with inner margin having 4–5 simple setae (7–8 in *C. watlingi*), and (2) uropod peduncle with inner margin bearing one sub-distal micro-serrate seta with single sub-terminal medial setule (3–6 in *C. watlingi*).

Males of the new species can be differentiated from males of *C*. *watlingi* by: (1) antennule peduncle articles 1–2 sub-equal in length (article 2 shorter than article 3 in *C. watlingi*), (2) antennule accessory flagellum slightly longer than basal article of main flagellum (shorter in *C. watlingi*), (3) uropod peduncle 5.6 times as long as wide (6.5 in *C. watlingi*), and (4) uropod exopod article 1 twice as long as wide (1.4 times in *C. watlingi*).

Only two other vaunthompsoniine genera, *Picrocuma* Hale, 1936 and *Pseudopicrocuma* Akiyama, 2012 from southwest and northwest Pacific, respectively, have reduced thoracic exopods and males with clasping, geniculate antennae, and lack pleopods. In this respect they appear superficially similar to *Carinacuma* and *Spilocuma*. We consider, however, that the similarities of these geographically separated genera are unrelated and are due to homoplasy. One apparently important systematic character, which supports this view is the distantly different morphology of the uropods of the Atlantic genera, which have uniarticulated endopods, while those from the Pacific are biarticulated. Further, *Carinacuma* and *Spilocuma* have vestigial exopods on pereopod-4, which are absent on *Picrocuma* and *Pseudopicrocuma*. Although, these two unrelated Atlantic and Pacific taxa exhibit geniculate-clasping antennae, the specific morphological details of these appendages are distinctively different. The male of *Carinacuma umesi* **sp. nov.** is distinguished from those of the other vaunthompsoniine species, which lack pleopods, in the following identification key.

### Key to the known males lacking pleopods within Vaunthompsoniinae

1. Antenna flagellum with five or six articles. Pereopod 4 without exopod. Uropod endopod uni-articulated (Figs. 5A–15E) …2

–Antenna flagellum with 10+ articles. Pereopod 4 having reduced or vestigial exopod. Uropod endopod bi-articulated (Figs. 15I–15K) …5

2. Antennule main flagellum composed of four articles. Uropod rami longer than peduncle (Fig. 15A) …*Pseudopicrocuma japonicum* (Akiyama, 2012)

–Antennule main flagellum composed of one or two articles. Uropod rami sub-equal to or shorter than peduncle length (Figs. 15B, 15D–15E) …3

3. Uropod peduncle broad, about twice as long as wide (Fig. 15B) …*Picrocuma crudgingtoni* Taft & Greenwood, 1996

–Uropod peduncle slender, at least three times as long as wide (Figs. 15D–15E) …4

4. Carapace length shorter than pereon length (Fig. 15G). Carapace-pereon length longer than pleon length (Fig. 15G) …*Picrocuma poecilotum sensu* (Tafe & Greenwood, 1996)

–Carapace length sub-equal to pereon length (Fig. 15H). Carapace-pereon length shorter than pleon length (Fig. 15H) …*Picrocuma rectangularis* Muhlenhardt-Siegel, 2003

5. Uropod endopod article 1 inner margin bearing 14 serrate setae (Fig. 15I). Uropod endopod article 2 inner margin bearing four serrate setae (Fig. 15I) …*Spilocuma salomani* Watling, 1967

–Uropod endopod article 1 inner margin bearing 5–6 serrate setae (Figs. 15J –15K). Uropod endopod article 2 inner margin bearing one serrate seta (Figs. 15J–15K) …6

6. Antennule: peduncle article 2 shorter than article 3 (Fig. 15L); accessory flagellum shorter than basal article of main flagellum (Fig. 15L) …*Carinacuma watlingi* (Omholt & Heard, 1979), **comb. nov.**

–Antennule: peduncle articles 2–3 sub-equal length (Fig. 15M); accessory flagellum slightly longer than basal article of main flagellum (Fig. 15M) …*Carinacuma umesi* **sp. nov.**

Hale (1936) created the monotypic genus *Picrocuma,* to receive the new species, *P. poecilotum* based on an ovigerous female holotype from Wynyard, Fossil Reef, Tasmania and in the same publication reported subadult specimens that he attributed to this species from Sellick’s beach Reef, Gulf St. Vincent’s Bay (South Australia). Later Hale (1945) described an adult male attributed to *P. poecilotum* based on a specimen from Table Bay, Tasmania, and reported a new northeastern range extension for this species to Myora Bight, Moreton Bay, Queensland. Besides its larger size and a geographical distance of over 1,600 km, there appear to be no major specific differences between the ovigerous female holotype of *P*. *poecilotum* and those examined by Tafe & Greenwood (1996) from Moreton Bay (see Tafe & Greenwood, 1996). Conversely, due to several morphological incongruencies between the original description of the male of *P*. *poecilotum* from Table Bay (Tasmania, (Hale, 1945)) and from Tangalooma (Moreton Bay, (Tafe & Greenwood, 1996)) such as: (1) the length of the carapace *v* s pereon length (longer in *P*. *poecilotum* from Tasmania (Fig. 15F) and shorter in *P*. *poecilotum* from Moreton Bay (Fig. 15G)), (2) the length of the carapace-pereon *vs* pleon length (shorter in *P*. *poecilotum* from Tasmania (Fig. 15F) and longer in *P*. *poecilotum* from Moreton Bay (Fig. 15G)), (3) the uropod shape (broadened in *P*. *poecilotum* from Tasmania (Fig. 15C) and slender in *P*. *poecilotum* from Moreton Bay (Fig. 15D)), and (4) the length of the uropod exopod *vs* uropod endopod length (shorter in *P*. *poecilotum* from Tasmania (Fig. 15C) and sub-equal in *P*. *poecilotum* from Moreton Bay (Fig. 15D)); which indicates that the male of *P*. *poecilotum* described by Hale (1945) from Table Bay (Tasmania) is not conspecific and appears to represent an undescribed species (Tafe & Greenwood, 1996). Until adult males attributed to *P*. *poecilotum sensu* Hale (1945) are examined and compared in detail, and, if feasible, a molecular study can be conducted to determine if *P*. *poecilotum* populations from both areas of Australia are conspecifics, the taxonomic status of this species remains unresolved. For these reasons, morphological features from the male of *P*. *poecilotum* (p. 473; fig. 68–F) presented by Tafe & Greenwood (1996) were used.

## Discussion

During a previous study by Morales-Núñez & Chigbu (2016), *Carinacuma umesi* **sp. nov**. was misidentified as *Spilocuma watlingi*, in MCB. This record now represents the first confirmed occurrence of *C. umesi* on the East Coast of North America. With the description of *C. umesi*, three of the seven vaunthompsoniine species with males lacking pleopods now occur in the northwest Atlantic (Fig. 2).

According to Omholt & Heard (1979), *Carinacuma watlingi* occurs most commonly in shallow-water with sand substrata adjacent to low energy barrier island beaches. A similar situation was found for *C. umesi* collected from Maryland Coastal Bays and associated barrier-island systems. Therefore, it is likely that members of the genus *Carinacuma* are generally restricted to subtidal, shallow-water in fine sandy substrata along protected beaches adjacent to the inlets of coastal bays and estuaries.

The discovery of *Carinacuma umesi* is the result of a more intensive fine (0.5 mm) sieving screening effort and more careful examination of the smaller coastal marine invertebrates in MCBs. Further, the small size of *C*. *umesi*, in conjunction with lack of taxonomic expertise might have led to their being overlooked or misidentified in previous benthic studies of these mid-Atlantic coast bays by other investigators (Llansó, Scott & Kelley, 2002; Llansó, Scott & Kelley, 2003; Llansó, Scott & Kelley, 2004; Llansó, Scott & Kelley, 2005; Llansó, Scott & Kelley, 2006; Llansó & Dew, 2010; Llansó, 2015). For the same reason, it would not be surprising if the geographical range of *C. umesi*, currently known from the MCBs, extends southward along the coast of the South Atlantic Bight (SAB).

## Conclusion

*Carinacuma***gen. nov.**, which is represented by two species, *C*. *umesi* and *C*. *watlingi*, is endemic to East and Gulf coasts of North America and appear to have no systematic affinities to the superficially similar western Pacific genera *Picrocuma* and *Pseudopicrocuma*. It has its closest systematic relationship with the North American genera *Spilocuma* and *Mancocuma*.

Heretofore the lack of published records for *Carinacuma umesi* from the East Coast of North America might have been due to the artifacts of sampling (e.g., sieve mesh-sizes too large to retain small species), and lack of taxonomic expertise to identify poorly known invertebrate taxa in the region.

##  Supplemental Information

10.7717/peerj.11740/supp-1 Supplemental Information 1 Abiotic information

## Abbreviations

MCBs: Maryland Coastal Bays
GoM: Gulf of Mexico
UMES: University of Maryland Eastern Shore
USNM: National Museum of Natural History, Smithsonian Institution, Washington DC
mm: millimeters
TBL: Total body length
PSU: Practical Salinity Unit
DO: Dissolved Oxygen
SE: standard error

## Additional Information and Declarations

### Data Availability

The following information was supplied regarding data availability: Abiotic factors are available in the Supplemental File.

### New Species Registration

The following information was supplied regarding the registration of a newly described species: Publication LSID: urn:lsid:zoobank.org:pub:3DF884AE-08A7-4CDD-B1D4-A7675ADC7F92 Carinacuma gen. nov. LSID: urn:lsid:zoobank.org:act:9F70BBDA-C25C-4F79-AC1D-B87D60A25C63 Carinacuma umesi sp. nov. LSID: urn:lsid:zoobank.org:act:A13E4A2B-8695-4649-88B6-82DF80F62C7F.
